# Single‐Cell RNA Editing Identifies T Cell ADAR1 as a Key Regulator of Immune Exhaustion and Anti‐PD‐1 Resistance in Colorectal Cancer

**DOI:** 10.1002/advs.76143

**Published:** 2026-06-15

**Authors:** Da Kang, Song‐Zuo Xie, Yong‐Zhou Luo, Xin‐Pei Deng, Xi‐Rong Tan, Ze‐Geng Chen, Ling‐Xing Zeng, Gong Chen, Pei‐Rong Ding, Zhi‐Zhong Pan, Rong‐Xin Zhang

**Affiliations:** ^1^ Department of Colorectal Surgery Sun Yat‐sen University Cancer Center Guangzhou Guangdong Province P. R. China; ^2^ State Key Laboratory of Oncology in South China, Guangdong Key Laboratory of Nasopharyngeal Carcinoma Diagnosis and Therapy, Guangdong Provincial Clinical Research Center For Cancer Sun Yat‐sen University Cancer Center Guangzhou Guangdong Province P. R. China; ^3^ Department of Nuclear Medicine Sun Yat‐sen University Cancer Center Guangzhou Guangdong Province P. R. China; ^4^ Department of Breast Oncology Sun Yat‐sen University Cancer Center Guangzhou Guangdong Province P. R. China; ^5^ Department of Urology Sun Yat‐sen University Cancer Center Guangzhou Guangdong Province P. R. China; ^6^ Department of Intensive Care Unit Sun Yat‐sen University Cancer Center Guangzhou Guangdong Province P. R. China; ^7^ Department of Clinical Laboratory, The First Affiliated Hospital, Jiangxi Medical College Nanchang University Nanchang Jiangxi P. R. China

**Keywords:** ADAR1, colorectal cancer, immunotherapy, T cell exhaustion, TGF‐β signaling pathway

## Abstract

ADAR1‐mediated RNA editing has been implicated in tumor immune evasion, primarily through tumor‐intrinsic interferon (IFN) signaling. However, its cell‐type‐specific roles within immune compartments, particularly T cells, remain unclear in colorectal cancer (CRC). RNA editing landscapes were profiled using bulk RNA sequencing and full‐length single‐cell RNA sequencing. ADAR1 expression and RNA editing activity were analyzed across the tumor microenvironment (TME), followed by functional validation and multi‐cohort clinical evaluation. Single‐cell analyses revealed elevated ADAR1 activity in tumor‐infiltrating T cells, defining an exhausted and proliferative T cell state associated with immune dysfunction. Functional experiments demonstrated that ADAR1 promotes T‐cell exhaustion and impairs cytotoxic activity. In vivo adoptive transfer models further confirmed that ADAR1 overexpression in T cells limits antitumor efficacy. Mechanistically, ADAR1 activated the TGF‐β‐SMAD signaling pathway. Clinically, elevated ADAR1 expression in T cells was associated with reduced response to anti‐PD‐1 therapy across immunotherapy cohorts. These findings identify ADAR1 as a key regulator of dysfunctional T cell states in CRC and suggest that targeting ADAR1 activity in T cells may represent a promising strategy for improving immunotherapy efficacy and developing predictive biomarkers.

## Graphical abstract

Single‐cell RNA editing analysis identifies ADAR1 as a regulator of dysfunctional T cell states in colorectal cancer. Elevated ADAR1 activity promotes T cell exhaustion and impairs antitumor immunity partly through TGF‐β‐SMAD signaling, contributing to anti‐PD‐1 resistance and highlighting T cell ADAR1 as a potential therapeutic target and biomarker.

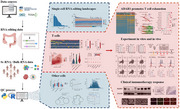

## 1 Introduction

Colorectal cancer (CRC) remains one of the most prevalent malignancies worldwide, characterized by an increasing metastatic incidence and a decreasing age of onset, posing a threat to public health [1, 2]. Traditional treatments, including chemotherapy, radiotherapy, and surgery, have long been the standard of care [3], while emerging immunotherapies have demonstrated long‐term remission in selected cases [4, 5, 6, 7]. The activation of tumor‐infiltrating immune cells requires the exposure of neoantigens, making high tumor mutational burden (TMB) a critical marker for immunotherapy response [8, 9]. Consequently, patients with microsatellite instability‐high (MSI‐H) or deficient‐mismatch‐repair (dMMR) tumors are more likely to benefit from immune checkpoint inhibitors (ICIs). However, only 15% of CRC patients exhibit these molecular features, and even some patients still fail to respond [10]. These observations underscore the urgent need to elucidate the molecular mechanisms underlying immune dysfunction and to expand the applicability of immunotherapy in CRC.

RNA editing represents an important layer of epigenetic regulation, with the adenosine‐to‐inosine (A‐to‐I) editing being the most common [11, 12, 13]. Because inosine is interpreted as guanosine during sequencing, A‐to‐I editing events are commonly detected as A>G (and complementary T>C) substitutions in RNA‐seq data. This process is catalyzed primarily by the adenosine deaminase acting on RNA 1 (ADAR1), which binds double‐stranded RNA (dsRNA) and converts base adenosine (A) to inosine (I), thereby influencing RNA stability, splicing, and translation [14, 15]. In tumor cells, disruptions in nucleic acid metabolism and chronic inflammation lead to the accumulation of dsRNA, which can be sensed by the melanoma differentiation‐associated gene 5 (MDA5), leading to activation of interferon (IFN) signals to exert the anti‐tumor effects [16, 17, 18, 19]. As an interferon‐stimulated gene (ISG), ADAR1 regulates tumor biology through two major mechanisms: first, ADAR1 edits specific coding regions of target transcripts, directly altering corresponding nucleotide sequences and protein products [20, 21]; second, and more importantly, ADAR1 prevents the activation of the dsRNA‐MDA5 axis, limiting downstream IFN signaling [22, 23]. Through these complementary functions, ADAR1 has been implicated in tumor immune evasion and resistance to immunotherapy [24].

With the expanding clinical application of ICIs, increasing attention has been paid to the role of ADAR1 in shaping immunotherapy response. Ishizuka et al. demonstrated that the loss of ADAR1 in melanoma could overcome immunotherapy resistance via the IFN pathway [25]. Similarly, there are also studies reported that the sensitivity to ADAR1 loss was associated with chronic inflammatory signaling [26, 27]. In addition, the non‐canonical functions of ADAR1 have also been gradually elucidated in the context of tumorigenesis and cancer treatment. For example, a recent study showed that activation of IFN signals could induce cell death in non‐ADAR1‐dependent tumors [28, 29]. Such necroptosis was potentially driven by Z‐form nucleic acids (Z‐DNA and Z‐RNA) and their sensors, such as Z‐DNA binding protein 1 (ZBP1), illustrating a potential mechanism of ADAR1 in the unresponsiveness to ICIs [30, 31, 32]. Meanwhile, the direct impacts of ADAR1 on coding genes have also been reported in various solid tumors (e.g., breast, ovarian, gastric etc.), suggesting ADAR1's implication in oncogenesis and potential for therapeutic targeting [33, 34, 35, 36, 37].

Notably, most existing studies investigating ADAR1 in cancer have predominantly focused on tumor cells or bulk tumor tissues, thereby obscuring cell‐type‐specific contributions within the tumor microenvironment (TME), particularly those of immune cells. Given that immune checkpoint blockade directly targets T cells, understanding how ADAR1 functions within distinct immune cell populations is critical for elucidating mechanisms of immunotherapy resistance. However, a systematic characterization of RNA editing landscapes and ADAR1 activity across different cellular compartments of the CRC remains lacking.

In this study, we performed an integrated analysis of RNA editing profiles using both bulk RNA sequencing and full‐length single‐cell RNA sequencing (scRNA‐seq) data to delineate ADAR1 expression and RNA editing activity in CRC. Our analyses reveal that ADAR1 expression in T cells represents an important and previously underappreciated component associated with T cell exhaustion and impaired responses to immunotherapy. By further integrating functional assays, in vivo validation, and multi‐cohort immunotherapy analyses, we demonstrate that ADAR1 activity in T cells is linked to distinct dysfunctional transcriptional programs, including activation of immunosuppressive signaling pathways such as TGF‐β signaling, and contributes to immune heterogeneity and resistance to ICIs in CRC.

## 2 Methods

### 2.1 Data Sources and Processing

Full‐length scRNA‐seq FASTQ files of 590 CRC cells were obtained from the European Genome‐phenome Archive Dataset (EGA Dataset ID: EGAD00001002727) [38]. The dataset comprised predominantly epithelial cells (∼68%), along with T cells (∼9%) and other cell types (∼22%), including B cells, macrophages, fibroblasts, and endothelial cells. The raw FASTQ files were quality‐controlled using fastp (v0.23.0), then mapped to the human genome assembly GRCh38 using Tophat2 (v2.1.1) to generate BAM files. Specific parameter settings were as follows: read‐edit‐dist was set to 3, and read‐realign‐edit‐dist was set to 0. The human genome annotation file from Gencode (v36, https://www.gencodegenes.org/human/) was used for downstream analyses.

Bulk RNA‐seq BAM files of 175 CRC samples (28 rectal cancers, 103 colon cancers, 44 tissues) were downloaded from The Cancer Genome Atlas (TCGA) dataset, and the detailed data processing workflow has been described previously. The clinical characterizations of the TCGA CRC patients were downloaded from UCSC Xena platform (https://xenabrowser.net/datapages/).

Three CRC scRNA‐seq datasets (GSE205506, GSE236581, and GSE132465) were collected from the Gene Expression Omnibus (GEO) database [39, 40, 41]. In the GSE205506 cohort, patients were divided into pathological complete response (pCR) and non‐pCR groups based on response to ICI therapy, and this cohort was used to observe dynamic changes in gene expression before and after treatment. The GSE236581 cohort was incorporated as an additional public immunotherapy cohort to validate the association between T cell ADAR1 expression and therapeutic response, including complete response (CR), non‐CR, tumor regression grade (TRG), and tumor regression ratio. All gene expression matrices of scRNA‐seq were independently analyzed using the Seurat package (v5.0.1) in R (v4.4.1). Count data were integrated into Seurat objects using the reciprocal Principal Component Analysis (RPCA) algorithm. Then, cells were filtered based on UMIs (>400 and <20 000 UMIs), features (>500 and <5000 features), and mitochondrial gene content (Epithelial cells <0.7 and other cells <0.2). After normalizing and scaling the data, principal component analysis (PCA) (resolution = 0.2, 20 PCs) and t‐distributed stochastic neighbor embedding (t‐SNE) were performed to subcluster cell subgroups based on cell‐specific markers. To ensure consistency across datasets, we referred to the original subgroup classifications where available and made minor adjustments according to our unified analysis workflow. Cell subpopulations were annotated based on markers from previous studies to maintain consistency.

### 2.2 Detection of RNA Editing Sites

REDItools2 (v1), a published tool for RNA editing detection, was used in this study to identify specific editing sites in the BAM files [42]. Due to the differences in RNA‐seq types, we applied corresponding filtering parameters: for scRNA‐seq data (‐q 25, ‐men 3, ‐me 3) and for bulk RNA‐seq data (‐q 25, ‐men 5, ‐me 3). Identified sites that overlapped with sites in dbSNP (v151) or COSMIC (v98) were considered potential tumor single‐nucleotide polymorphisms (SNPs) and were excluded. Sites covered with reads ≥10 and editing frequency ≥ 0.1 were included in the subsequent analysis. A similar processing workflow was applied to the TCGA bulk data. The detected sites were then annotated using various methods, including snpEff (v5.1), the UCSC RepeatMasker annotation file, and the REDIportal annotation file, to elucidate the region types for downstream analysis.

### 2.3 Identification of Differential Editing Sites (DESs)

Similar to the identification of differentially expressed genes in RNA‐seq count analysis, differential RNA editing sites (DESs) are utilized to observe editing differences within samples, such as between tumors and normal tissues. In this study, two approaches were employed to detect these DESs: (i) Wilcoxon rank‐sum test was used to assess the statistical significance of editing frequency differences across samples; (ii) the REDIT‐LLR tool (v1), based on the log‐likelihood ratio method, was implemented to identify DESs [43]. This approach has been previously published and validated for its robustness. RNA editing sites identified by either method with an FDR‐adjusted *p* value <0.05 and an editing frequency >0.1 were considered DESs.

### 2.4 Calculation of Alu Editing Index (AEI)

The Alu Editing Index (AEI) is a weighted score used to assess global RNA editing levels, particularly focusing on the activity of ADAR1 within Alu elements. This method has been reported in previous studies as a reliable measure of overall RNA editing activity [44]. The principle behind AEI is that virtually all editing activity by ADAR1 occurs in Alu elements. We applied this score to evaluate the average editing levels in both scRNA‐seq and bulk‐seq data. The specific calculation steps were as follows: (i) extracted all A‐to‐G (T‐to‐C) reads within Alu elements; (ii) calculated the ratio of the number of G reads to all extracted reads (sum of A reads and G reads) as the AEI in the sample.

### 2.5 RNA Single‐Base Substitution (RNA‐SBS) Signatures

RNA single‐base substitution (RNA‐SBS) signatures were calculated to analyze the contribution of RNA editing to tumor heterogeneity. RNA editing bases and the two adjacent bases' information were collected to create a matrix involving the proportions of types [45]. This approach achieved an exponential enhancement in feature diversity through combinatorial expansion from the original 12 editing types to 192 context‐specific categories. Then, the R package NMF (v0.28) was used to cluster the matrix, and the Brunet method was applied with 50 iterations to ensure robust clustering. The optimal k value was determined by evaluating dispersion and silhouette scores, resulting in four RNA‐SBS types, and these types were characterized across different cell subtypes.

### 2.6 RNA Stability Prediction

To explore the potential impact of RNA editing on mRNA folding structure, the RNA folding prediction tool Mfold web server was utilized [46]. This study focused on genes that exhibited differences both in editing sites and gene expression, under the hypothesis that RNA editing could influence gene expression by altering RNA structure. To verify this point, for each editing site, its 100 bp flanking nucleotides were extracted and then fed into Mfold to obtain the most energetically favorable RNA secondary structures based on free energy. The free energy of the RNA structures with and without editing was compared. A negative free energy difference (ΔG < 0) indicated that RNA editing potentially stabilized the RNA structure.

### 2.7 Gene Set Enrichment Analysis (GSEA)

Enrichment analysis across different cell types was performed using the R package clusterProfiler (v4.12) based on the gene sets obtained from MSigDB (v7.5.1). Additionally, this study included two immune gene signatures derived from published studies: exhausted signatures (including genes LAG3, TIGIT, PDCD1, HAVCR2, CTLA4) and cytotoxic signatures (including genes IL2, GZMA, GNLY, PRF1, GZMB, GZMK, IFNG, NKG7). Single‐sample gene set enrichment analysis (ssGSEA) was used to quantify function‐specific scores in both scRNA‐seq and bulk RNA‐seq data by the “ssgseaParam” function from the R package GSVA (v1.52.3). The ssGSEA scores were then analyzed statistically using either the Wilcoxon rank‐sum test or Pearson correlation test with other variables (R packages rstatix and linkET). To address redundancy and ensure reproducibility in the enrichment terms, we utilized the R package simplifyEnrichment (v1.14.1) to cluster similar terms. Additionally, the Metascape database (https://metascape.org/) was used to validate the reliability of our analysis.

### 2.8 Immune Cell Infiltration Scores

Quantification of immune cell infiltration in TCGA bulk data was estimated via R package IBOR (v2.0.0). CIBERSORT, TIMER, xCell, ESTIMATE, EPIC, and quanTIseq were used to calculate the immune scores in the samples. These scores were compared with different clinical characterizations and used for downstream analysis.

### 2.9 Survival Analysis

Overall survival (OS) time and status data were obtained from the UCSC Xena platform. The survival curves were generated using the R packages survival (v3.7) and survminer (v0.5). The Kaplan‐Meier method or log‐rank test was utilized to compare survival significance between different patient groups. To illustrate the general impact of gene expression on survival, the Kaplan‐Meier plotter (https://kmplot.com/analysis/) was employed for further exploration.

### 2.10 Principal Component Analysis (PCA)

To explore the underlying structure of the RNA editing profiles and reduce its dimensionality, principal component analysis (PCA) was performed with the R package factoextra (v 1.0.7). Based on this model, patients from the TCGA‐CRC data were divided into three distinct groups for downstream analysis to explore potential biological differences.

### 2.11 Cell‐Cell Communication Analysis

The R package CellChat (v1.6.1) based on the cellular ligand‐receptor pairs (L–R pairs) was employed to analyze intercellular communication at the single‐cell level. The intercellular communication probabilities were calculated with a normalized Seurat object and the “CellChatDB.human” dataset. The potential interaction strengths and enriched ligand‐receptor interactions were used to analyze global communication patterns and signal networks mediated by different L‐R pairs. To explore the differences in ligand‐receptor interactions and signaling pathways, scRNA‐seq data were divided into subgroups according to the expression of specific genes.

### 2.12 Pseudo‐Timing Analysis of Cell Trajectory

To investigate changes in CD8+ T cell pseudotime‐dependent gene expression, the R package Monocle3 (v1.3.7) was utilized to infer cell trajectories. The processed Seurat object was imported to explore potential cellular differentiation pathways. The annotated CD8+ T cells were ordered to construct an explicit principal graph from the scRNA data. Lineage trajectory plot of genes in T cells was generated in a pseudotime‐dependent manner to better resolve complex biological processes.

### 2.13 Isolation of Immune Cells and Cell Line Culture

Peripheral blood samples were collected from both healthy donors and CRC patients. Peripheral blood mononuclear cells (PBMCs) were isolated following standard procedures, such as storage in anticoagulated tubes (BD, Cat# 367841), dilution with PBS, spreading over Ficoll (Cytiva, Cat# 17144003), and centrifugation using a density gradient. After lysing erythrocytes, CD3+ T cells were isolated using CD3 MicroBeads (Miltenyi, Cat# 130‐097‐043) and cultured in X‐VIVO medium (LONZA, Cat# 04–418Q) containing 1000 IU/mL IL‐2. Subsequently, ImmunoCult CD3/CD28 human T cell activator (STEMCELL, Cat# 10971) was added at a concentration of 25 µL/mL to stimulate the T cells for 24 h. The HEK293T cells and the human CRC cell lines HCT116 were obtained from the American Type Culture Collection (ATCC). All cells were authenticated using short tandem repeat (STR) testing and were cultured in RPMI‐1640 (Invitrogen) supplemented with 10% fetal bovine serum (Gibco) and Penicillin‒streptomycin (Gibco, Cat# 15240062). Mycoplasma status kit (Vazyme, Cat# D101) was used monthly to ensure the cultures were free from contamination.

### 2.14 Plasmids and Transfection

The ADAR1 overexpression plasmids were generated by inserting coding sequences (ADAR1‐p110 and ADAR1‐p150 coding sequences) into pCDH‐CMV‐MCS‐EF1‐copGFP empty plasmid, and the sgRNA targeting ADAR1 was added to the U6‐Cas9‐EGFP empty plasmid. Lentiviral infection protocols were performed to obtain ADAR1‐overexpression and ADAR1‐knockout T cells. Pre‐stimulated T cells were infected at an MOI of 10 in the presence of 8 µg/mL polybrene with lentivirus. Half of the medium was replaced in 24 h, and then cells were cultured for an additional 24 h before assessing the transduction efficiency by flow cytometry.

### 2.15 Flow Cytometry

For surface markers staining: T cells were resuspended in 100 µL of PBS, followed by the staining of Fixable Viability Stain 780 (1:1000, BD, Cat# 564996), FITC‐CD3 (1:100, BioLegend, Cat# 344804), BV421‐PD1 (1:100, BD, Cat# 562516), Brilliant Violet 605 (1:100, BioLegend, Cat# 372712)/PE (1:100, BioLegend, Cat# 372704)‐TIGIT, and Alexa Fluor 647 (1:100, BD, Cat# 565716)/BV421 (1:100, BD, Cat# 565720)‐LAG3 to the cell suspension. The mixture was incubated on ice for 30 min, centrifuged, and resuspended in 200 µL of PBS. The stained cells were then analyzed by flow cytometry.

*For intercellular marker staining*: T cells were treated with Cell Activation Cocktail (with Brefeldin) (BioLegend, Cat# 423303) for 5 h and then resuspended in PBS. Fixable Viability Stain 780 and PE/Cyanine7‐CD3 (1:100, BioLegend, Cat# 300316) were added to the cell suspension and incubated on ice for 30 min. After washing once with PBS, cells were fixed and permeabilized using the Fixation/Permeabilization Kit (BD, Cat# 554714). Subsequently, intracellular antibodies PE‐TNFα (BioLegend, Cat# 502909), APC‐IFNγ (BioLegend, Cat# 502512), FITC‐perforin (BioLegend, Cat# 308104), and PC5.5‐Granzyme B (BioLegend, Cat# 372212) were added to the cell suspension at a dilution of 1:100. The mixture was incubated on ice for 30 min, centrifuged, and resuspended in 200 µL of PBS. The cells were then analyzed by flow cytometry.

### 2.16 Enzyme‐Linked Immunosorbent Assay (ELISA)

The secretion of the type I/II interferon (IFN) in T cell culture supernatants was examined using commercial ELISA kits (Abcam, Cat# ab264610, ab236895) following the manufacturer's guidelines.

### 2.17 Lactate Dehydrogenase (LDH) Release Assay

Approximately 1 × 104 HCT116 cells were seeded into 96‐well plates and incubated overnight. Subsequently, 3 × 104 T cells with different treatments were added to each well and co‐cultured for 24 h to induce tumor cell killing, then the culture supernatant was collected for analysis. LDH is originally present in the cytoplasm and is released into the culture medium following cell death. LDH was quantified using the CytoTox 96 Non‐Radioactive Cytotoxicity Assay (Promega, Cat# G1780) according to the manufacturer's protocol.

### 2.18 Apoptosis Assay

HCT116 cells (5 × 105/well) were seeded into 24‐well plates for incubation overnight. T cells with different quantities (5 × 105, 1.5 × 106, and 5 × 106) were added to induce tumor cell apoptosis for another 24 h. Subsequently, we labeled the HCT116 cells using the Annexin V‐Elab Fluor 647/DAPI apoptosis kit (Elabscience, Cat# E‐CK‐A254‐100), and detected apoptosis rates with flow cytometry.

### 2.19 In Vivo Tumor Model and Adoptive T Cell Transfer

Female NSG mice (6 weeks old) were used for all in vivo experiments. To establish a subcutaneous tumor model, HCT116 cells (1 × 106 cells in 200 µL PBS) were subcutaneously injected into the flank of each mouse on day 0. Tumor growth was monitored by measuring tumor length and width, and tumor volume was calculated using the formula: 0.5 × length × width^2^. For adoptive T cell therapy, human T cells with different ADAR1 perturbations were administered intravenously via tail vein injection every four days after tumor implantation. Each mouse received 5 × 106 T cells per injection. T cells were assigned to one of the following groups: saline control, negative control T cells, ADAR1‐p150 T cells, or ADAR1‐sg T cells. Mice were sacrificed on day 20 after tumor implantation. Tumors were excised, weighed, and processed for downstream analyses. All animal procedures were approved by the Experimental Animal Ethics Committee of Sun Yat‐sen University Cancer Center (L102042025070D).

### 2.20 Western Blotting Assay (WB)

T cells of CRC patients were lysed using RIPA buffer (Proteintech, Cat# PR20001) to obtain total protein. The processed proteins were separated using SDS‒PAGE (EpiZyme) and subsequently transferred onto PVDF membranes (Merck Millipore). The blocking membranes were then incubated with anti‐Phospho‐Smad2(S465+S467)/ Phospho‐Smad3(S423+S425) (Wanleibio, Cat# WL02305, 1:1000), anti‐Phospho‐Smad2/3 (Thr8) (Affinity, Cat# AF3367, 1:1000), anti‐Smad2/3 (Wanleibio, Cat# WL01520, 1:1000), anti‐ADAR1 (SANTACRUZ, Cat# sc‐271854, 1:1000) and anti‐β‐actin (CST, Cat# 3700S, 1:2000) antibodies at 4°C for 12 h, while HRP‐conjugated secondary antibodies (Abcam, Cat# ab6789, 1:4000) at room temperature for 1 h. The expression levels of proteins were quantified via chemiluminescence.

### 2.21 Real‐Time Quantitative PCR Assay (qRT‐PCR)

Total RNA was extracted using the TRIzol reagent (Thermo Fisher, Cat# 15596018), and then reverse‐transcribed into complementary DNA (cDNA) using the HiScript III RT SuperMix (Vazyme, Cat# R323‐01). The cDNA was subsequently amplified using the qPCR SYBR Green Master Mix (Yeasen, Cat# 11201ES08). Relative gene expression was calculated using the 2‐ΔΔCT method. The primer sequences were detailed below:

ADAR1 Forward: 5’‐CTGAGACCAAAAGAAACGCAGA‐3’

ADAR1 Reverse: 5’‐GCCATTGTAATGAACAGGTGGTT‐3’

ACTB Forward: 5’‐CATGTACGTTGCTATCCAGGC‐3’

ACTB Reverse: 5’‐CTCCTTAATGTCACGCACGAT‐3’

### 2.22 Immunohistochemistry Assay (IHC)

The sections of paraffin‐embedded tissues were deparaffinized (65°C for 1 h) and rehydrated (alcohol for 5 min and ddH_2_O for 5 min). The antigens were extracted using sodium citrate buffer and heat‐induced retrieval. The samples were then blocked with 5% fetal bovine serum to prevent nonspecific binding after incubation with 3% H_2_O_2_ for 15 min. The tissues were incubated with primary antibodies (anti‐ADAR1, SANTACRUZ, Cat# sc‐271854, 1:100) at 4°C for 12 h, followed by incubation with HRP‐conjugated secondary antibodies (BOSTER, Cat# SA1020) at room temperature for 1.5 h. The sections were ultimately stained with DAB (BOSTER, Cat# SA1020) and analyzed as digital images through scanning.

### 2.23 Immunofluorescence Staining (IF)

To analyze the tumor tissue sections, the sections were deparaffinized, rehydrated in ethanol, and blocked in Triton X‐100 (MCE, Cat# HY‐Y1883A) buffer. Then, the primary antibodies (anti‐ADAR1, SANTACRUZ, Cat# sc‐271854, 1:100; anti‐CD3, CST, Cat# 78588, 1:100) were incubated with the tissues for 12 h at 4°C, followed by incubation with the corresponding secondary antibody at room temperature for 1.5 h. The sample images were observed with scanning software after washing and DAPI staining.

### 2.24 Statistical Analysis

All statistical analyses were conducted using R (v4.4.1) and GraphPad Prism (v9.5.1), and the data were presented as mean ± standard deviation. The analyses in this study were performed after assessing normality and frequency tests on the data. The *p‐*value was calculated using *t*‐test or the Wilcoxon rank sum test for comparisons between two groups and ANOVA for multiple groups in numeric variables, while the Chi‐square test was used for clinical parameters in categorical variables. Survival analysis was conducted using the Kaplan‐Meier method or the log‐rank test. Receiver operating characteristic (ROC) analysis was performed to evaluate the ability of T cell ADAR1 expression and exhaustion scores to distinguish CR from non‐CR patients. The *p*‐value or FDR‐adjusted *p*‐value less than 0.05 indicated statistical significance.

## 3 Results

### 3.1 Single‐Cell RNA Editing Landscapes Reveal Heterogeneity in Colorectal Cancer

**FIGURE 1:**
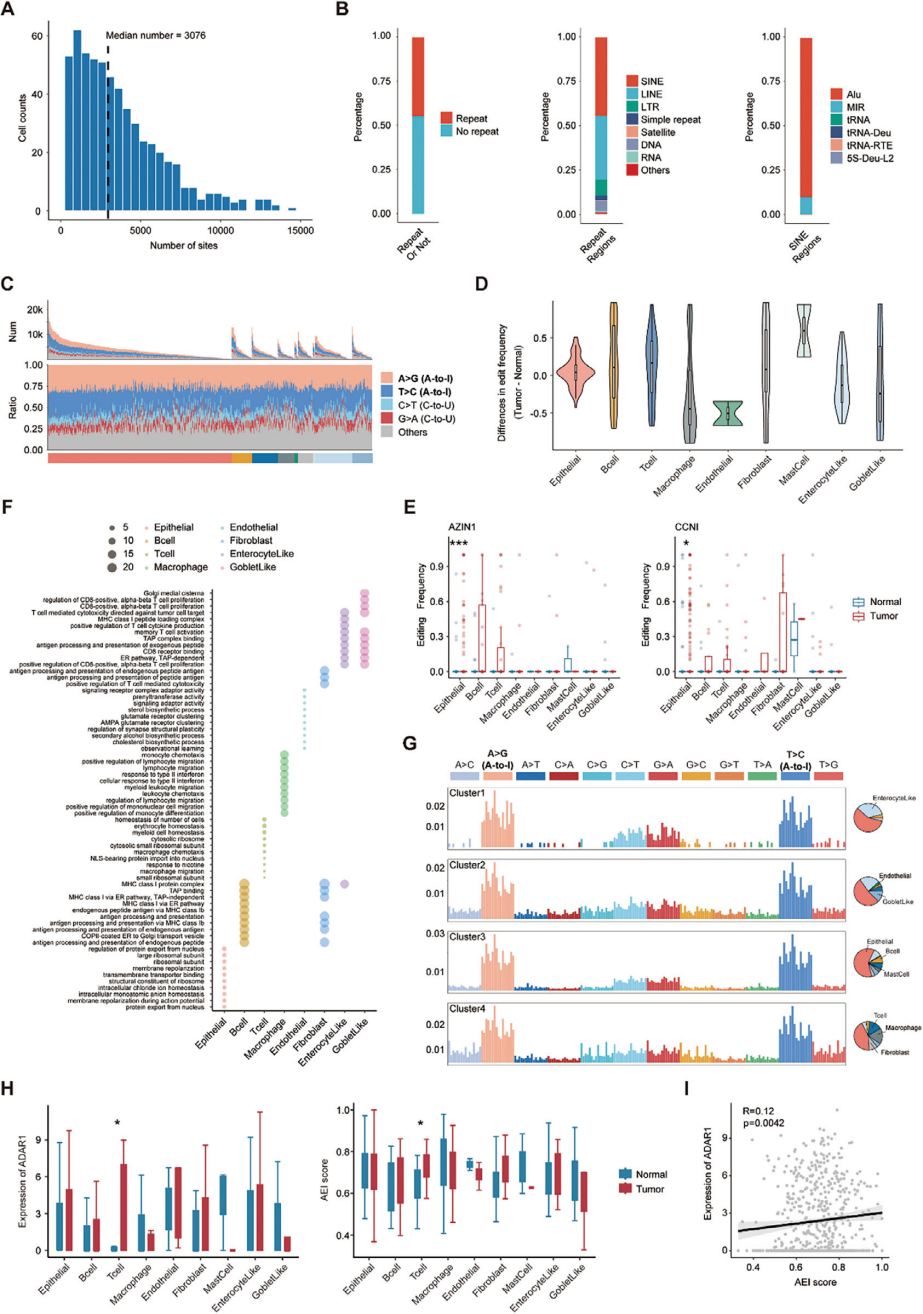
Single‐cell RNA editing landscapes reveal heterogeneity in colorectal cancer. (A) Distribution of the number of RNA editing sites per cell across all single cells, with the median value indicated by a dashed line (median = 3076 sites). (B) Genomic annotation of RNA editing sites, showing their distribution across repetitive and non‐repetitive regions, major repeat classes, and SINE subfamilies. (C) Number and relative proportion of different RNA editing types identified from single‐cell RNA editing profiles. (D) Differences in RNA editing frequencies between tumor and normal cells across major cell types. (E) Editing frequencies of representative genes (e.g., AZIN1 and CCNI) across different cell types and tissues. (F) Functional enrichment analysis of edited genes, showing the top‐enriched terms across different cell types. (G) Trinucleotide context distribution of RNA‐SBS signatures identified by unsupervised clustering, with pie charts indicating the cellular composition of each cluster. (H) ADAR1 expression levels and AEI across different cell types in normal and tumor tissues. (I) Correlation between ADAR1 expression and AEI at the single‐cell level. *, *p*‐value <0.05; **, *p*‐value <0.01; ***, *p*‐value <0.001; ****, *p*‐value <0.0001.

To characterize RNA editing events across different cell types in CRC, we analyzed the full‐length scRNA‐seq data from the EGAD00001002727 cohort. At single‐cell resolution, the A‐to‐I (A>G, T>C) and cytidine to uridine (C‐to‐U; C>T and G>A) substitutions emerged as the predominant RNA editing types, exhibiting distinct chromosomal enrichment patterns (Figure S1A). Similar patterns were observed in TCGA‐CRC bulk RNA‐seq data, supporting the robustness of the identified profiles (Figure S1B). Across all cells, the median number of editing sites was 3076 (Figure 1A). These sites were primarily located in non‐coding and repetitive regions, with a high proportion (44%) mapping to short interspersed nuclear elements (SINEs), particularly Alu elements (90%) (Figure 1B and Figure S1C). Motif analysis revealed sequence preferences consistent with the known binding motifs of ADAR1 and APOBEC3A (the C‐to‐U editing writer enzyme) [47]. As expected, global editing frequencies correlated positively with the expression levels of ADAR1 and APOBEC3A (Figure S1D,E).

Differential editing site (DES) analysis across cell and tissue types showed that A‐to‐I events remained the dominant category in both abundance and proportion (Figure 1C and Table S1). Compared with normal tissues, tumors exhibited a modest global increase in editing frequency; however, the distribution of these changes varied markedly across cell types (Figure 1D). In addition, editing events in AZIN1, CCNI, and HNRNPK were also observed (Figure 1E and Figure S1F); these genes have been previously reported as oncogenic targets [48, 49, 50]. Functional enrichment analysis of edited genes further highlighted cell‐type‐specific biological processes, with immune‐related interaction terms enriched in T cells and macrophages (Figure 1F). Unsupervised clustering of RNA single‐base substitution (RNA‐SBS) signatures identified four distinct editing patterns, dominated by A‐to‐I substitutions but differing in cellular composition (Figure S1G). Cluster 4 was enriched for immune cells, particularly T cells and macrophages (Figure 1G), consistent with the functional enrichment analysis. Given the high prevalence of A‐to‐I events, we compared ADAR1 expression levels and the Alu Editing Index (AEI) to assess global RNA editing activity. The results showed that T cells exhibited significantly elevated ADAR1 expression and AEI, exceeding those observed in epithelial cells (Figure 1H). Correlation analysis further revealed a positive association between AEI and ADAR1 expression, indicating that ADAR1 activity is tightly coupled with overall RNA editing intensity (Figure 1I).

Collectively, these single‐cell‐resolution analyses demonstrate pronounced heterogeneity in RNA editing landscapes across cell types in CRC, with T cells exhibiting particularly elevated ADAR1‐associated editing activity.

### 3.2 Bulk Transcriptomic Analyses Link ADAR1‐Associated Immune Signals to T Cells

**FIGURE 2:**
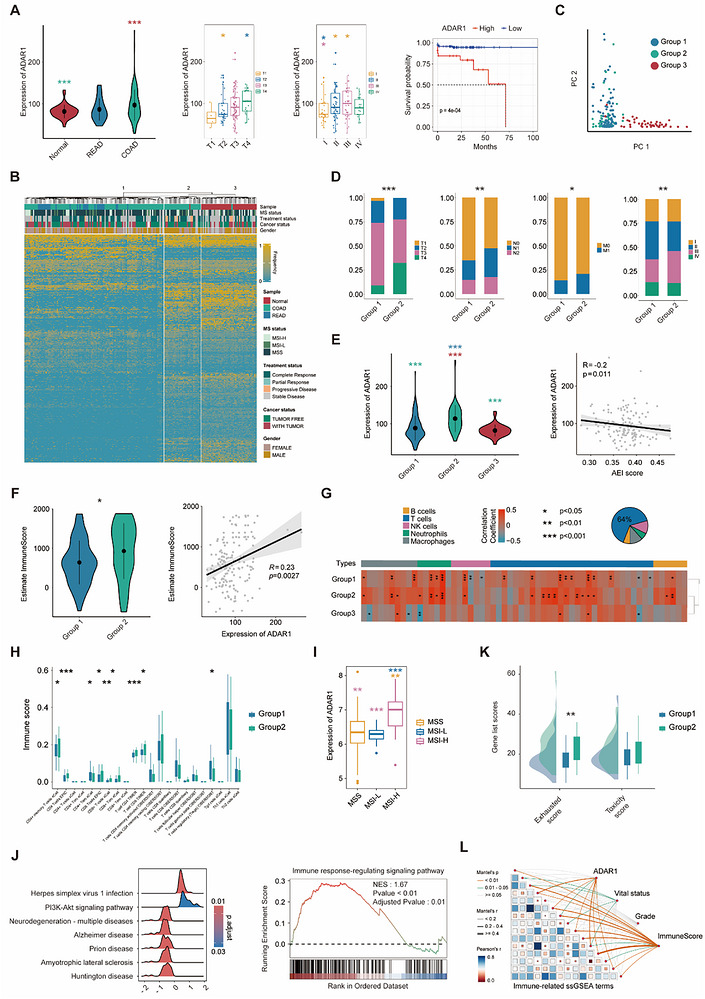
Bulk transcriptomic analyses link ADAR1‐associated immune signals to T cells. (A) ADAR1 expression levels in normal and tumor tissues, stratified by tumor type and clinical stage, and Kaplan‐Meier analysis of overall survival (OS) in the TCGA‐CRC cohort. (B) Heatmap of RNA editing frequencies across TCGA‐CRC samples, with unsupervised clustering identifying three distinct editing‐based groups. (C) PCA of TCGA‐CRC samples based on RNA editing profiles, showing separation among the three groups. (D) Distribution of clinical characteristics, including tumor stage, lymph node status, metastasis status, and pathological stage, across clustering groups. (E) ADAR1 expression levels across the three RNA editing‐defined groups and the correlation between ADAR1 expression and the AEI. (F) ESTIMATE immune scores across clustering groups and correlation between immune scores and ADAR1 expression. (G) Correlation analysis between ADAR1 expression and immune cell‐associated signatures inferred by multiple deconvolution algorithms, with a pie chart summarizing the relative immune cell composition differences between group 2 and group 1. (H) Comparison of T cell subtype‐associated scores between clustering groups. (I) ADAR1 expression levels stratified by microsatellite status (MSS, MSI‐L, and MSI‐H). (J) Pathway enrichment analysis of ADAR1‐associated genes identified from TCGA‐CRC bulk transcriptomic data. (K) Comparison of T cell functional scores, including exhaustion and cytotoxicity signatures, between clustering groups. (L) Mantel test, and correlation analyses illustrating associations between ADAR1 expression, immune‐related ssGSEA terms, and clinical or immune features. *, *p*‐value < 0.05; **, *p*‐value < 0.01; ***, *p*‐value < 0.001; ****, *p*‐value < 0.0001.

To further investigate cell‐type‐specific RNA editing patterns in bulk tissue profiles, we analyzed ADAR1 expression and clinical characteristics in the TCGA‐CRC cohort. ADAR1 expression was significantly elevated in tumor tissues and was associated with more advanced clinical stage and poorer overall survival (OS) (Figure 2A). Across samples, differentially edited coding genes showed a generally inverse relationship between editing frequency and transcript abundance, with enrichment in RNA metabolism‐related pathways (Figure S2A). Based on bulk RNA editing profiles, all samples were classified into three groups (Table S2), with group 2 enriched for microsatellite instability (MSI) tumors and group 3 representing normal tissues (Figure 2B). Principal component analysis (PCA) revealed clear separation among these groups (Figure 2C). Consistent with this stratification, clinical characteristics showed that patients in group 2 exhibited more advanced disease stages than those in group 1 (Figure 2D). Group 2 samples displayed higher ADAR1 expression but lower global AEI, revealing an inverse correlation between total ADAR1 abundance and bulk RNA editing levels (Figure 2E).

Given the single‐cell observations described above, we hypothesized that variation in immune cell infiltration might contribute to this apparent discrepancy. Indeed, immune infiltration analysis using ESTIMATE scores demonstrated that group 2 tumors exhibited lower tumor purity and higher immune scores, both of which correlated positively with ADAR1 expression (Figure 2F and Figure S2B). Deconvolution analyses using multiple immune inference algorithms, including xCell, EPIC, TIMER, CIBERSORT, and quanTIseq, consistently revealed that ADAR1 expression correlated most strongly with T cell‐associated signatures in group 2 samples (Figure 2G). Further subpopulation analysis indicated that both CD4^+^ and CD8^+^ T cell fractions contributed to this association (Figure 2H). Consistent with enhanced immune infiltration, MSI‐H tumors exhibited elevated ADAR1 expression compared with microsatellite‐stable tumors (Figure 2I).

Together, these analyses indicate that ADAR1‐associated transcriptional signals observed in bulk CRC tissues are closely linked to immune cell‐related programs, with T cells representing a principal cellular context. Enrichment analysis of ADAR1‐associated genes revealed pathways related to antiviral responses and immune regulation (Figure 2J). Furthermore, single‐sample GSEA (ssGSEA) demonstrated that higher ADAR1 expression was associated with increased T cell exhaustion scores and reduced cytotoxic signatures (Figure 2K and Figure S2C), along with positive correlations with immune response‐related gene sets and exhaustion markers (Figure 2L and Figure S2D).

### 3.3 Single‐Cell Profiling Reveals ADAR1‐Associated Remodeling of T Cell Exhaustion

**FIGURE 3:**
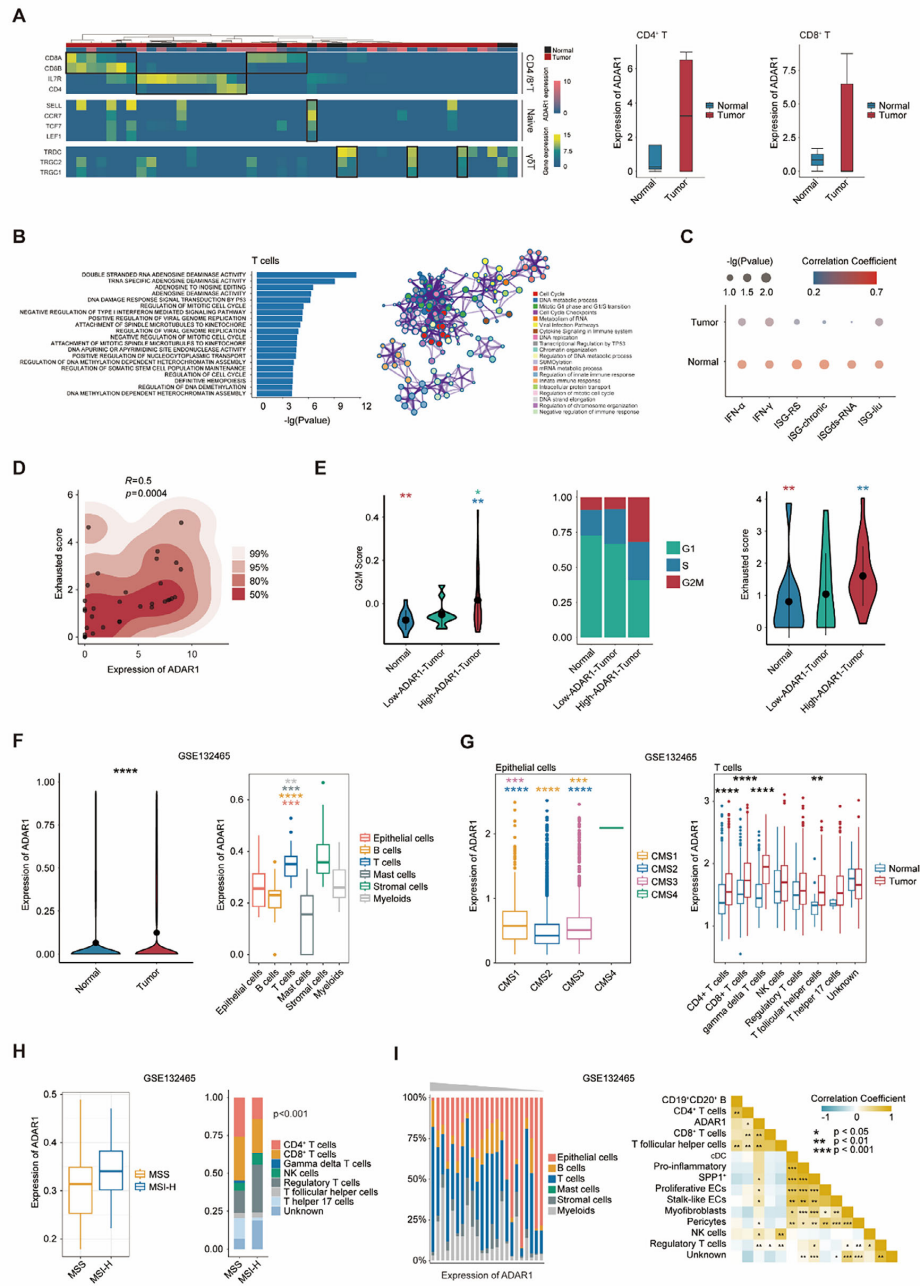
Single‐cell profiling reveals ADAR1‐associated remodeling of T cell exhaustion. (A) Heatmap showing the expression of representative T cell markers and ADAR1 across single T cells, along with comparison of ADAR1 expression levels between normal and tumor‐infiltrating CD4^+^ and CD8^+^ T cells. (B) Functional enrichment analysis of genes co‐expressed with ADAR1 in T cells. (C) Correlation analysis between ADAR1 expression and IFN‐related signatures in T cells from normal and tumor tissues. (D) Spearmen correlation analysis between ADAR1 and the exhausted score of T cells. (E) Cell‐cycle state distribution and exhaustion scores in T cells stratified by ADAR1 expression levels. (F) ADAR1 expression levels across tissues and major cell lineages in the GSE132465 scRNA‐seq dataset. (G) Comparison of ADAR1 expression levels among epithelial cell subtypes and T cell subpopulations in the GSE132465 dataset. (H) ADAR1 expression levels and immune cell composition stratified by microsatellite status (MSS versus MSI‐H) in the GSE132465 dataset. (I) Proportions of major cell types across patients ranked by ADAR1 expression and correlation analysis between ADAR1 expression and immune cell subsets. *, *p*‐value < 0.05; **, *p*‐value < 0.01; ***, *p*‐value < 0.001; ****, *p*‐value < 0.0001.

To resolve the functional heterogeneity underlying ADAR1‐associated immune signals observed in bulk CRC tissues, we performed subclustering analyses of T cell populations based on scRNA‐seq profiles. ADAR1 expression was increased in both CD4^+^ and CD8^+^ T cells within tumor tissues, consistent with observations from bulk analyses (Figure 3A and Figure S3A). Gene co‐expression analysis demonstrated that ADAR1‐high T cells were enriched for transcriptional programs related to the negative regulation of immune responses and RNA metabolic processes (Figure 3B). Given the established role of ADAR1 in IFN signaling, we further examined the relationship between ADAR1 expression and IFN response signatures. This association was attenuated in tumor‐infiltrating T cells (Figure 3C), suggesting altered IFN‐associated regulatory activity in the TME. The ssGSEA showed that ADAR1 expression was positively correlated with exhaustion‐related signatures but not with cytotoxicity scores (Figure 3D and Figure S3B). In parallel, cell‐cycle analysis revealed that ADAR1‐high T cells exhibited increased proliferative activity, characterized by an elevated proportion of cells in the G2/M phase (Figure 3E). These findings suggest that ADAR1 may contribute to the exhausted T cell phenotype through dual mechanisms: promoting T cell exhaustion and facilitating the expansion of this dysfunctional T cell subpopulation.

To validate these observations in an independent dataset with greater cellular coverage, we analyzed the scRNA‐seq dataset GSE132465. Consistent with the initial analysis, ADAR1 expression was elevated in tumor tissues and T cells (Figure 3F and Figure S3C). Within epithelial cells, CMS1 and CMS3 subtypes exhibited higher ADAR1 expression, which are characterized by distinct immune and chromosomal instability features (Figure 3G). Stratification by ADAR1 expression further revealed that patients with higher ADAR1 levels, particularly those with MSI‐H tumors, exhibited increased expression of canonical exhaustion markers (Figure 3H and Figure S3D). We next ranked patients in descending order based on average ADAR1 expression to compare cell‐type proportions (Figure S3E). This analysis indicated a positive correlation between ADAR1 expression and the abundance of multiple T cell subtypes (Figure 3I). Functional enrichment analyses confirmed the associations between ADAR1 expression and both T cell exhaustion and proliferation, aligning with prior observations (Figure S3F).

In parallel, epithelial cells were analyzed separately (Figure S4A,B). ADAR1 expression was elevated in tumor epithelial cells and positively correlated with global RNA editing activity. Functional enrichment and ssGSEA analyses indicated suppression of interferon signaling and enrichment of protein metabolism‐related pathways (Figure S4C–E). Further interrogation of protein metabolism‐associated genes identified NAP1L1 as a highly edited and upregulated transcript in tumor epithelial cells (Figure S4F–H). RNA stability analysis suggested that RNA editing may enhance NAP1L1 mRNA stability, which was associated with poorer overall survival in CRC patients (Figure S4I,J).

Together, these single‐cell analyses demonstrate that ADAR1 expression delineates a distinct dysfunctional T cell state in CRC, characterized by immune exhaustion and enhanced proliferative activity, while exerting divergent regulatory effects in epithelial cells.

### 3.4 Immunotherapy Cohorts Link ADAR1‐Associated Exhausted T Cell States to Treatment Resistance

**FIGURE 4:**
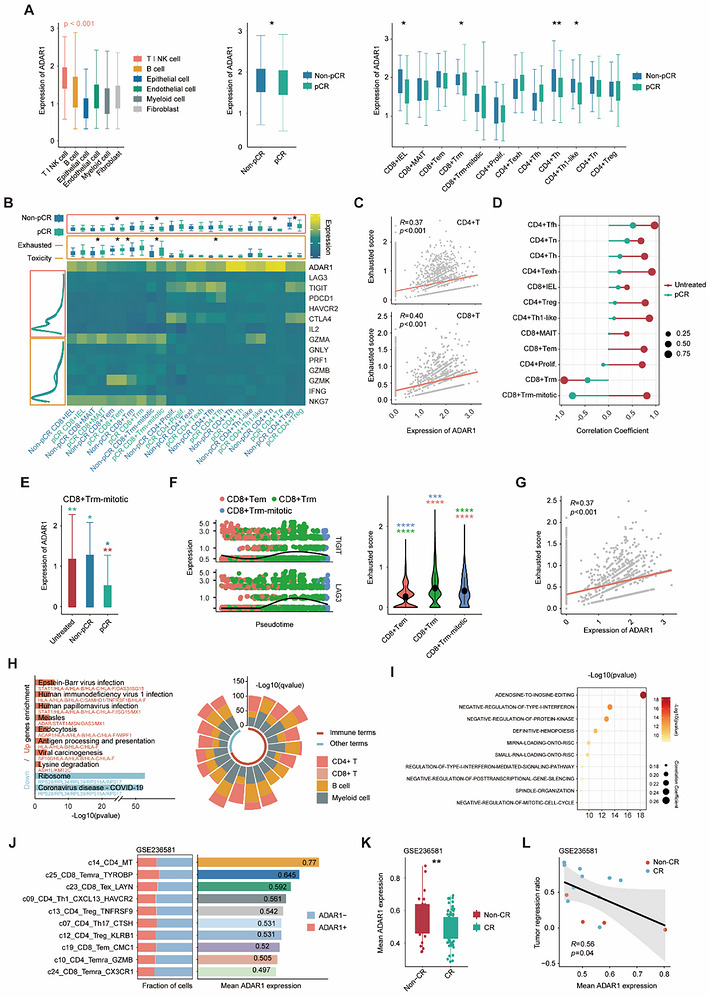
Immunotherapy cohorts link ADAR1‐associated exhausted T cell states to treatment resistance. (A) ADAR1 expression across major cell types and T cell subsets in CRC patients treated with anti‐PD‐1 therapy. (B) Heatmap showing the expression of exhaustion‐ and cytotoxicity‐associated markers in T cells from non‐pCR and pCR patients. Left, distribution of functional scores; top, summarized functional scores across groups. (C) Correlation analysis between ADAR1 expression and exhausted scores in T cells. (D) Lollipop plot illustrating changes in correlation coefficients between ADAR1 expression and the relative abundance of T cell subpopulations before treatment and in pCR patients. (E) ADAR1 expression levels in the CD8^+^ Trm‐mitotic subset across untreated, non‐pCR, and pCR groups. (F) Pseudotime trajectory analysis of CD8^+^ T cell subsets, showing the dynamic distribution of ADAR1 expression and exhaustion marker expression along differentiation trajectories. (G) Correlation analysis between ADAR1 expression and exhaustion scores in CD8^+^ T cells. (H) Pathway enrichment analysis of ADAR1‐associated gene programs across immune cell types. (I) ssGSEA analysis highlighting biological pathways associated with ADAR1 expression in immune cells. (J) Top ten T cell subpopulations with the highest mean ADAR1 expression in the GSE236581 cohort. Left bars show the fraction of ADAR1^+^ and ADAR1^−^ cells; right bars show mean ADAR1 expression. (K) Mean ADAR1 expression in T cells from non‐CR and CR patients in the GSE236581 cohort. (L) Spearman correlation between mean ADAR1 expression in T cells and tumor regression ratio in the GSE236581 cohort. *, *p*‐value < 0.05; **, *p*‐value < 0.01; ***, *p*‐value < 0.001; ****, *p*‐value < 0.0001.

To explore the clinical relevance of ADAR1‐associated T cell states, we first analyzed a scRNA‐seq dataset of CRC patients treated with anti‐PD‐1 therapy (GSE205506). Following quality control and cell‐type annotation (Figure S5A), ADAR1 expression was evaluated across immune compartments before and after immunotherapy. ADAR1 expression in T cells was significantly reduced after treatment, with the most pronounced decrease observed in patients achieving pathological complete response (pCR) (Figure 4A and Figure S5B). Consistently, functional state analysis revealed increased cytotoxic scores and decreased exhaustion scores in T cells from pCR patients (Figure 4B). ADAR1 expression in both CD4^+^ and CD8^+^ T cells showed strong positive correlations with exhaustion scores and with the relative abundance of dysfunctional T cell subsets (Figure 4C and Figure S5C). Comparative analyses of T cell subtype composition before and after ICI treatment demonstrated that the association between ADAR1 expression and most T cell subpopulations was markedly attenuated in pCR patients, indicating remodeling of ADAR1‐linked T cell states following effective immunotherapy (Figure 4D).

Among CD8^+^ T cell subsets, the proliferating CD8^+^ Trm‐mitotic population—previously reported as a negative factor for ICI efficacy—displayed a significant reduction in both frequency and ADAR1 association in pCR patients (Figure 4D,E). Cell trajectory analysis revealed progressive upregulation of exhaustion markers, including TIGIT and LAG3, with the CD8^+^ Trm‐mitotic subset occupying a terminal state characterized by high ADAR1 expression, elevated exhaustion scores, and increased G2/M phase activity (Figure 4F,G and Figure S5D,E). In parallel, cell‐cell interaction analyses demonstrated that ADAR1‐high T cells exhibited diminished interaction strength with other immune populations, including T cells, B cells, and myeloid cells (Figure S5F). Pathway enrichment analyses further associated ADAR1 expression with suppression of interferon signaling and modulation of ribosomal and cell‐cycle‐related programs (Figure 4H,I).

To further validate these findings, we incorporated an additional public immunotherapy cohort, GSE236581. After standardized processing and subclustering, diverse T cell populations were identified (Figure S5G). Consistent with GSE205506, ADAR1 expression was enriched in CD8^+^ T cells and CD4^+^ regulatory T cells (Figure S5H), with CD8^+^ Temra, CD8^+^ Tex, and multiple CD4^+^ Treg subsets among the top ADAR1‐expressing populations (Figure 4J). ADAR1 expression in T cells positively correlated with exhaustion scores (Figure S5I) and was higher in non‐CR than CR patients (Figure 4K). At the patient level, T cell ADAR1 expression negatively correlated with tumor regression ratio (Figure 4L). Functional enrichment of ADAR1‐high T cells further revealed interferon signaling and antigen presentation‐related programs (Figure S5J), consistent with the immune‐regulatory features observed in GSE205506.

Collectively, analyses of two immunotherapy cohorts indicate that ADAR1‐associated exhausted and proliferative T cell states are reproducibly linked to poor response to immune checkpoint blockade. These findings strengthen the clinical relevance of T cell ADAR1 activity and suggest that CRC patients with downregulated ADAR1 expression in T cells are more likely to benefit from ICI treatment.

### 3.5 ADAR1 Promotes T Cell Exhaustion and Impairs Antitumor Cytotoxicity In Vitro

**FIGURE 5:**
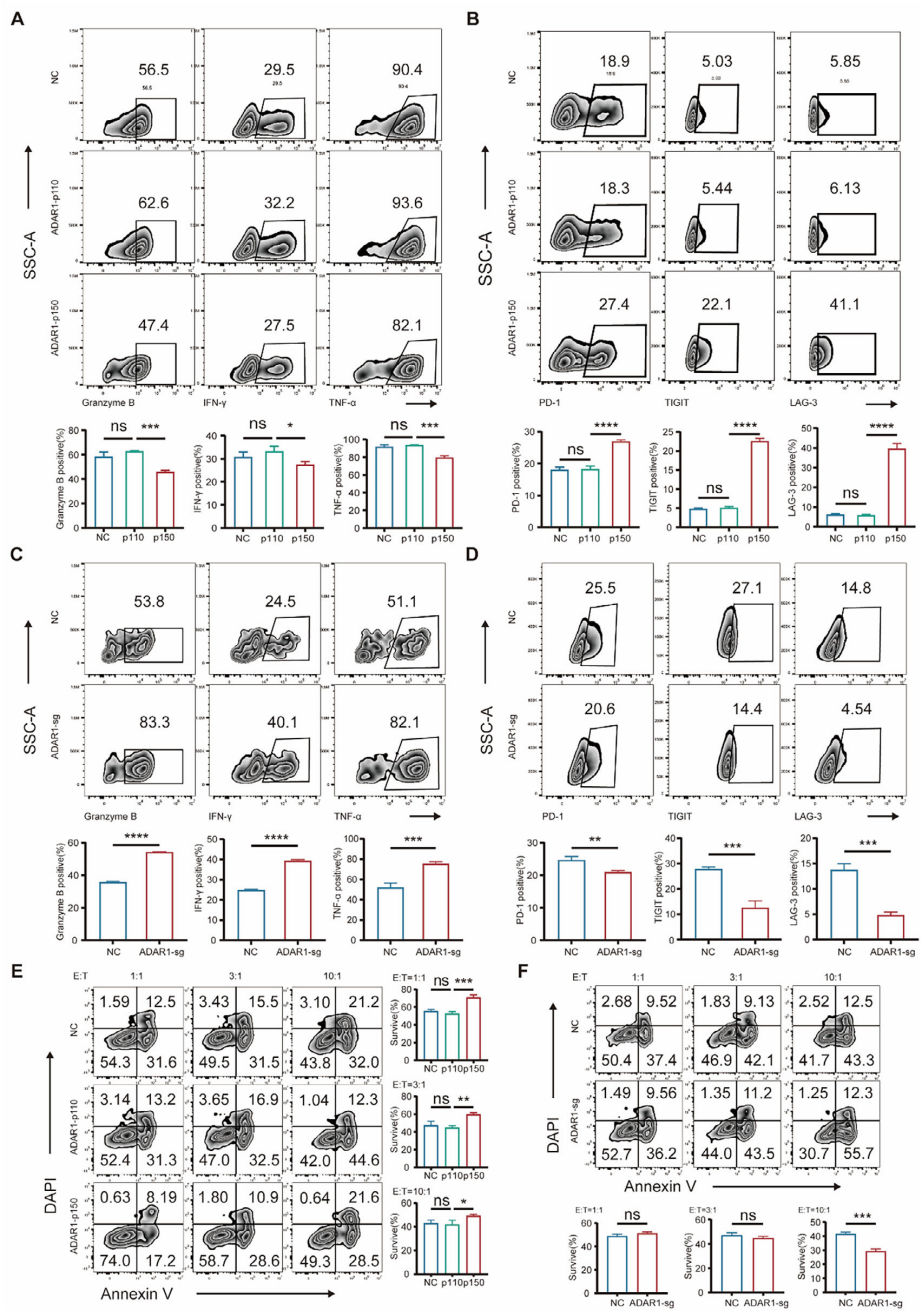
ADAR1 promotes T cell exhaustion and impairs antitumor cytotoxicity in vitro. (A) Flow cytometric analysis of effector molecules (Granzyme B, IFN‐γ, and TNF‐α) in human primary T cells overexpressing ADAR1 isoforms (ADAR1‐p110 or ADAR1‐p150) and negative control (NC) T cells. Representative plots (top) and quantification (bottom) are shown. (B) Flow cytometric analysis of inhibitory receptors (PD‐1, TIGIT, and LAG3) in ADAR1‐p110‐ and ADAR1‐p150‐overexpressing T cells compared with NC T cells. Representative plots and summary statistics are shown. (C) Flow cytometric analysis of effector molecule expression in ADAR1‐knockout (ADAR1‐sg) T cells and NC T cells. (D) Flow cytometric analysis of inhibitory receptor expression in ADAR1‐sg T cells compared with NC T cells. (E) Flow cytometry‐based apoptosis analysis (Annexin V staining) of HCT116 colorectal cancer cells cocultured with ADAR1‐p110‐ or ADAR1‐p150‐overexpressing T cells or NC T cells at indicated effector‐to‐target (E:T) ratios. (F) Apoptosis analysis of HCT116 cells cocultured with ADAR1‐sg or NC T cells at the indicated E:T ratios. *, *p*‐value < 0.05; **, *p*‐value < 0.01; ***, *p*‐value < 0.001; ****, *p*‐value < 0.0001.

To experimentally validate the functional impact of ADAR1 on T cell states, stable ADAR1‐modulated human primary T cells with ADAR1 overexpression (ADAR1‐p110 and ADAR1‐p150) or knockout (ADAR1‐sg) were generated (Figure S6A,B). Among the two isoforms, the IFN‐inducible ADAR1‐p150 exerted the most pronounced effects on T cell function. Overexpression of ADAR1‐p150 significantly increased the expression of inhibitory receptors, including PD‐1, TIGIT, and LAG3, while concomitantly reducing effector cytokine production (Figure 5A,B). In contrast, ADAR1‐deficient T cells exhibited reduced expression of exhaustion markers and enhanced cytotoxic signatures (Figure 5C,D).

Consistent with these phenotypic changes, ELISA assays of coculture supernatants demonstrated increased secretion of Granzyme B, IFN‐γ, and TNF‐α in ADAR1‐sg T cells compared with controls (Figure S6C,D). Functional cytotoxicity assays further showed that ADAR1 knockout enhanced T cell‐mediated killing of HCT116 CRC cells, whereas ADAR1‐p150 overexpression significantly impaired tumor cell apoptosis and LDH release (Figure 5E,F and Figure S6E,F).

Together, these results provide direct functional evidence that ADAR1 activity, particularly through the ADAR1‐p150 isoform, promotes T cell exhaustion and diminishes antitumor cytotoxic capacity, consistent with the dysfunctional T cell states identified at single‐cell resolution.

### 3.6 ADAR1 Overexpression in T Cells Limits Antitumor Efficacy In Vivo Through TGF‐β Signaling

**FIGURE 6:**
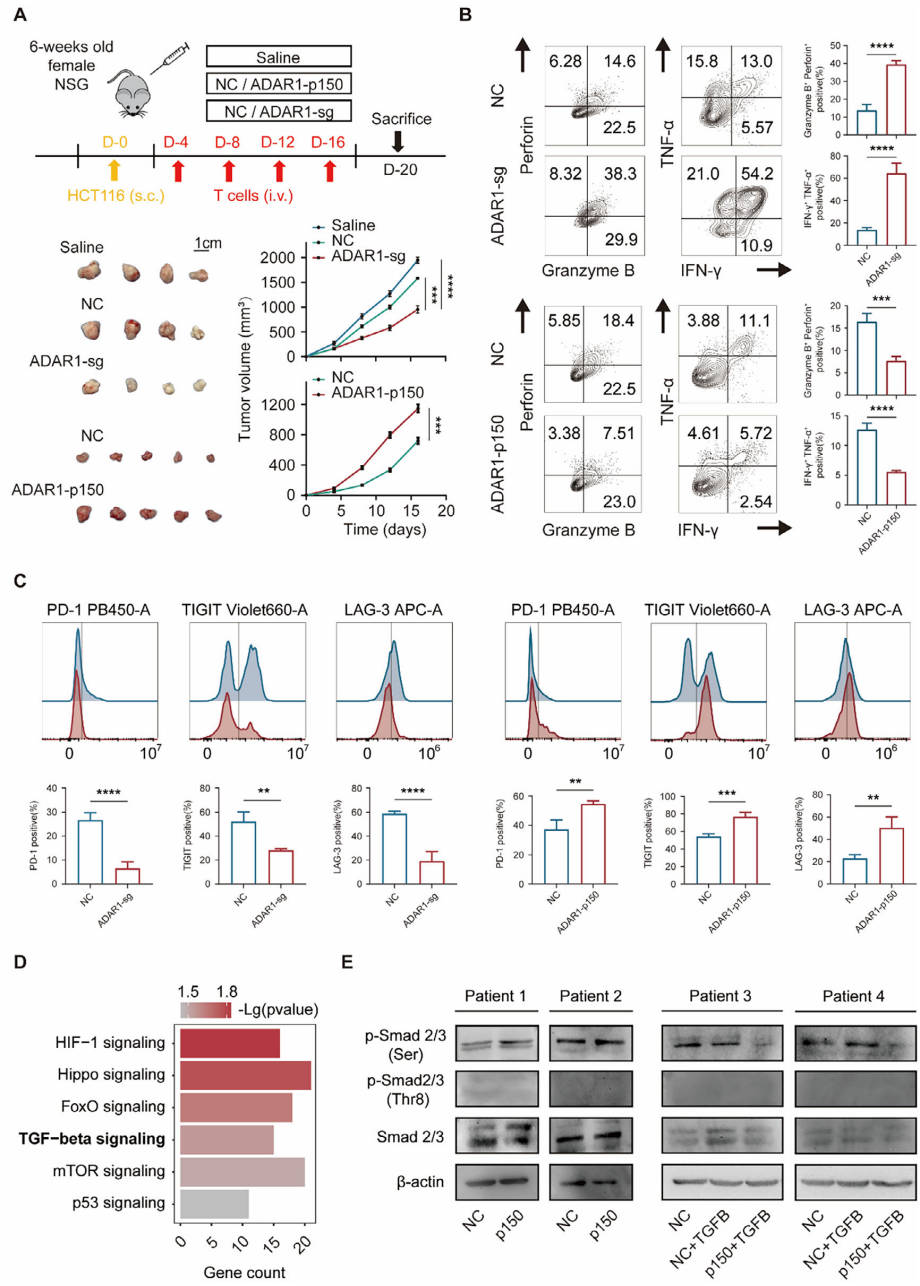
ADAR1 overexpression in T cells limits antitumor efficacy in vivo through TGF‐β signaling. (A) Schematic illustration of the in vivo adoptive T cell transfer experiment. Six‐week‐old female NSG mice were subcutaneously (s.c.) injected with HCT116 cells on day 0, followed by intravenous (i.v.) infusion of saline, negative control (NC) T cells, ADAR1‐p150‐overexpressing T cells, or ADAR1‐sg T cells every four days. Representative images of excised tumors and tumor growth curves are shown. (B) Representative flow cytometry plots showing Granzyme B, Perforin, TNF‐α, and IFN‐γ expression in tumor‐infiltrating T cells from mice receiving NC, ADAR1‐sg, or ADAR1‐p150 T cells. (C) Quantification of inhibitory receptor expression (PD‐1, TIGIT, and LAG3) on tumor‐infiltrating T cells from the indicated groups. (D) Pathway enrichment analysis of differentially expressed genes identified by RNA sequencing of NC and ADAR1‐p150‐overexpressing T cells, highlighting enrichment of the TGF‐β signaling pathway. (E) Immunoblot analysis of phosphorylated SMAD2/3 (Ser), phosphorylated SMAD2/3 (Thr8), total SMAD2/3, and β‐actin in NC and ADAR1‐p150‐overexpressing T cells, with or without treatment with a TGF‐β‐neutralizing antibody. *, *p*‐value < 0.05; **, *p*‐value < 0.01; ***, *p*‐value < 0.001; ****, *p*‐value < 0.0001.

To assess the impact of ADAR1 activity in T cells on antitumor immunity in vivo, we performed adoptive T cell transfer experiments (Figure 6A). Compared with saline controls, adoptive T cell transfer suppressed tumor growth, whereas mice receiving ADAR1‐p150 T cells exhibited accelerated tumor progression relative to NC controls. In contrast, transfer of ADAR1‐sg T cells resulted in enhanced tumor growth inhibition (Figure 6A).

Flow cytometric analysis of excised tumors further revealed reduced frequencies of Granzyme B‐, Perforin‐, TNF‐α‐, and IFN‐γ‐positive T cells in the ADAR1‐p150 group (Figure S6G), whereas ADAR1‐sg T cells displayed increased effector marker expression, consistent with enhanced cytotoxic function (Figure 6B). Analysis of inhibitory receptor expression showed that ADAR1‐p150‐overexpressing T cells exhibited markedly increased expression of PD‐1, LAG3, and TIGIT, whereas ADAR1‐sg T cells displayed reduced levels of these exhaustion‐associated markers, indicating that ADAR1 activity promotes T cell exhaustion in vivo (Figure 6C).

To explore the molecular basis of ADAR1‐mediated T cell dysfunction, we performed RNA sequencing of NC and ADAR1‐p150‐overexpressing T cells. Pathway analysis revealed significant enrichment of the TGF‐β signaling pathway in ADAR1‐overexpressing T cells (Figure 6D). Consistent with this transcriptional signature, western blot (WB) analysis demonstrated increased phosphorylation of SMAD2/3 at canonical serine residues in ADAR1‐p150 T cells, whereas phosphorylation at the non‐canonical Thr8 site remained unchanged, indicating preferential activation of the canonical TGF‐β‐SMAD signaling axis (Figure 6E). To functionally assess the involvement of TGF‐β signaling, we performed rescue experiments using a TGF‐β‐neutralizing antibody. Blockade of TGF‐β signaling markedly attenuated ADAR1‐induced SMAD2/3 phosphorylation at canonical serine residues (Figure 6E), supporting a functional link between ADAR1 activity and activation of the TGF‐β‐SMAD signaling axis.

Collectively, these results indicate that ADAR1 overexpression in T cells promotes immune exhaustion and limits antitumor efficacy in vivo, at least in part through activation of TGF‐β‐SMAD2/3 signaling.

### 3.7 Elevated ADAR1 Expression in T Cells Correlates With Poor Immunotherapy Response

In the GSE236581 cohort, T cell ADAR1 expression decreased after ICI treatment (Figure S7A), consistent with GSE205506, while higher proportions of ADAR1‐positive T cells were observed in patients with higher tumor regression grade (TRG) (Figure S7B). ROC analysis showed that T cell ADAR1 expression achieved a higher AUC than the exhaustion score for distinguishing CR from non‐CR patients (AUC = 0.875 vs. 0.65; Figure S7C), supporting its potential clinical relevance as an immunotherapy‐associated T cell marker.

**FIGURE 7:**
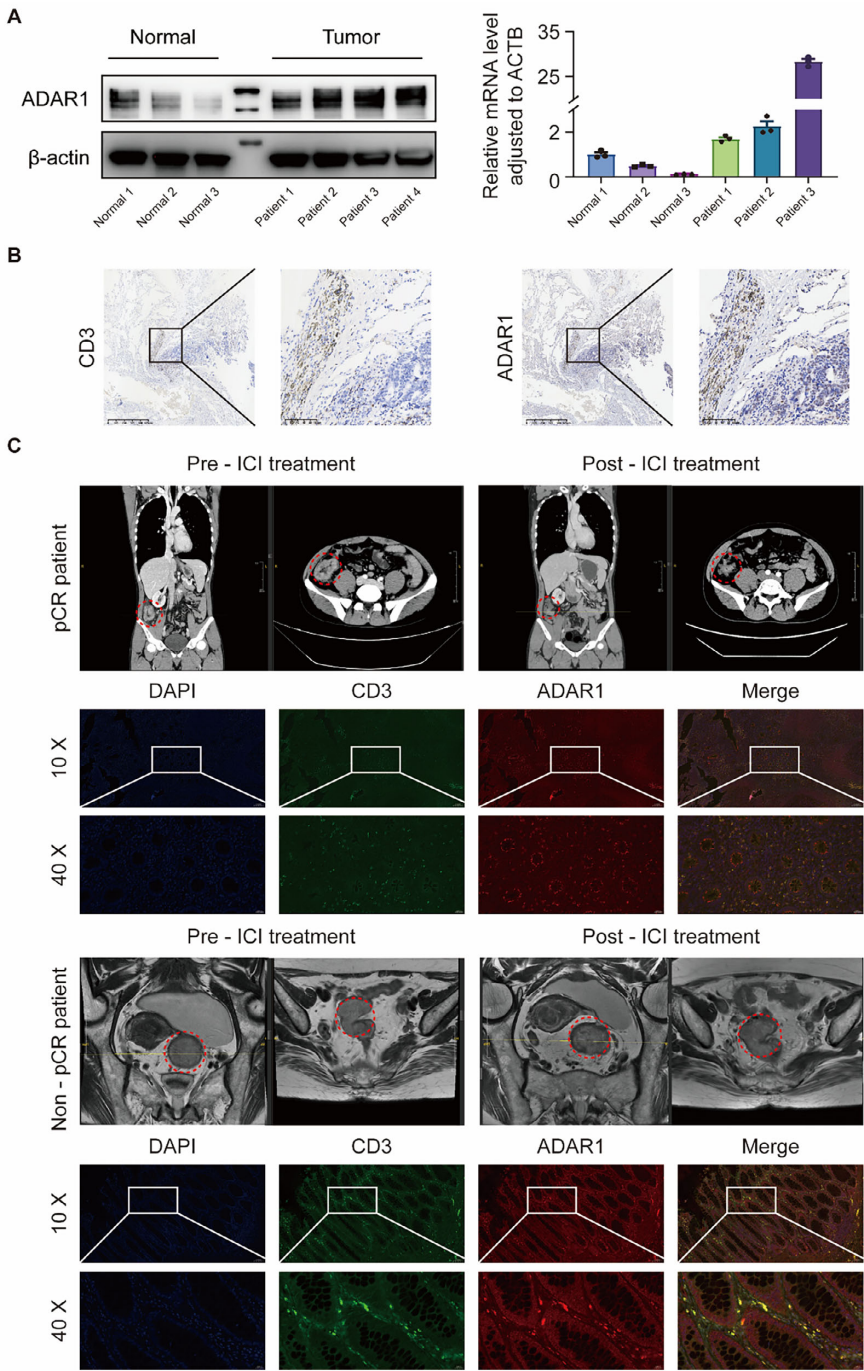
Elevated ADAR1 expression in T cells correlates with poor immunotherapy response. (A) WB and qRT‐PCR analyses of ADAR1 expression in peripheral blood T cells isolated from healthy donors and patients with CRC. (B) Representative IHC staining of CD3 and ADAR1 in CRC tumor tissues, showing spatial distribution and co‐localization of ADAR1 within CD3^+^ T cells. (C) Representative radiological images and multiplex immunofluorescence staining from CRC patients with ICI treatment. The upper images showed complete tumor regression in pCR patients, with no spatial co‐localization of ADAR1 and CD3. In contrast, the lower images depicted non‐pCR patients, where ADAR1 and CD3 were spatially co‐localized.

To assess ADAR1 expression in clinical samples, peripheral blood T cells were collected from healthy individuals and patients with CRC. Both WB and qRT‐PCR revealed increased ADAR1 expression at the protein and transcript levels in T cells derived from CRC patients (Figure 7A). Immunohistochemical (IHC) analysis of paired tumor sections further demonstrated spatial co‐localization of ADAR1 with the T cell marker CD3 within CRC tissues (Figure 7B and Figure S7D).

To further validate the association between T cell ADAR1 expression and immunotherapy response, tumor specimens from an independent cohort of CRC patients treated with anti‐PD‐1 therapy at SYSUCC were analyzed (Table 1). Post‐treatment imaging and pathological evaluations were used to assess therapeutic efficacy, with patients showing no residual disease classified as having achieved pCR. Patients who achieved pCR exhibited abundant T cell infiltration with minimal ADAR1 expression in CD3^+^ cells, whereas non‐pCR patients displayed pronounced co‐localization of ADAR1 and CD3 (Figure 7C and Figure S7E).

**TABLE 1:** Clinical characteristics of CRC patients undergoing ICI treatment in this study.

Samples	Treatment	Outcomes	Stage	CD3 (IF)	ADAR1 (IF)	Co‐localization (IF)
1	PD‐1	SD/PD	pT3N0	(+)	(+)	Yes
2	PD‐1	SD/PR	pT4N0	(+)	(+)	Yes
3	PD‐1	SD/PR	pT2N0	(+)	(+)	Yes
4	PD‐1	SD/PR	pT2N1	(+)	(+)	Yes
5	PD‐1	SD/PR	pT3N2	(+)	(+)	No
6	PD‐1	SD/PD	pT4N0	(+)	(+)	No
7	PD‐1	SD/PR	pT3N2	(+)	(−)	No
8	PD‐1	CR	pT0N0	(+)	(+)	No
9	PD‐1	CR	pT0N0	(+)	(−)	No
10	PD‐1	CR	pT0N0	(+)	(−)	No
11	PD‐1	CR	pT0N0	(+)	(−)	No

Collectively, these data support an association between elevated ADAR1 expression in T cells and poor response to immune checkpoint blockade. Together with evidence from the immunotherapy cohort, these findings suggest that ADAR1‐positive T cell states may serve as a clinically relevant indicator of dysfunctional antitumor immunity in CRC.

## 4 Discussion

In this study, we systematically characterized RNA editing landscapes in CRC by integrating bulk and scRNA‐seq data, revealing an important role for ADAR1 in shaping intra‐tumoral heterogeneity. Through comprehensive experimental validation, multi‐cohort immunotherapy analyses, and clinical specimen validation, we further demonstrated that ADAR1 expression defines an exhausted T cell phenotype associated with impaired antitumor immunity and resistance to immune checkpoint blockade.

Leveraging single‐cell‐resolution RNA editing profiles, we observed a significant increase in ADAR1 expression and global RNA editing activity in tumor‐infiltrating T cells. Distinct RNA‐SBS signatures and cell‐type‐specific editing patterns further underscored the context‐dependent functions of ADAR1 within the TME. At the tissue level, analysis of TCGA‐CRC data revealed that ADAR1 expression was positively associated with immune infiltration scores, suggesting that ADAR1‐associated signals are enriched within immune‐related programs. Importantly, functional analyses consistently demonstrated that ADAR1 expression defines an exhausted and proliferative T cell state, characterized by impaired effector function and enhanced negative immune regulatory programs. Mechanistically, our study provides evidence that ADAR1‐mediated T cell dysfunction is linked to activation of the TGF‐β signaling pathway. Transcriptomic profiling of ADAR1‐overexpressing T cells revealed significant enrichment of TGF‐β‐related pathways, which was further supported by increased phosphorylation of canonical SMAD2/3 serine residues. Functional blockade of TGF‐β signaling attenuated ADAR1‐associated SMAD2/3 activation, establishing a mechanistic connection between ADAR1 activity and engagement of the immunosuppressive TGF‐β‐SMAD axis. These observations were supported by both in vitro and in vivo experiments, in which modulation of ADAR1 activity directly altered inhibitory receptor expression, cytokine production, and the antitumor cytotoxic capacity of T cells. Across immunotherapy scRNA‐seq cohorts, we further observed that ADAR1‐associated dysfunctional T cell states were reproducibly linked to poor treatment response. In the GSE205506 cohort, the proliferative CD8^+^ Trm‐mitotic subset was enriched for ADAR1 expression, and both ADAR1 expression and its association with this dysfunctional subset were selectively diminished in patients achieving pCR but persisted in non‐responders. In the GSE236581 cohort, elevated ADAR1 expression in T cells was similarly associated with non‐CR status and reduced tumor regression ratio, further supporting the clinical relevance of ADAR1‐positive T cell states. Clinically, analysis of specimens from the ICI‐treated cohort revealed abundant T cell infiltration across tumors; however, pronounced ADAR1‐T cell co‐localization was observed in non‐responsive cases. Collectively, these results indicate that the functional state of tumor‐infiltrating T cells, rather than their abundance alone, is a critical determinant of immune microenvironment status and immunotherapy efficacy, positioning ADAR1 as a key regulator of T cell dysfunction in CRC.

Previous studies of ADAR1 in cancer have predominantly focused on tumor epithelial cells, in which ADAR1 regulates innate immune sensing, IFN signaling, and tumor cell function through RNA editing‐dependent mechanisms [22, 23, 24]. Seminal work by Ishizuka and Liu established a link between ADAR1‐mediated RNA editing and IFN signaling, highlighting its relevance to immune checkpoint blockade [25]. Subsequent studies by Gannon, Zhang [29, 30], and others further demonstrated that ADAR1 influences tumor cell fate and promotes tumorigenesis by editing coding transcripts involved in proliferation, therapeutic resistance, and metastasis [33, 34, 35, 36, 37]. Consistent with these reports, our analyses confirmed elevated ADAR1 expression and enhanced RNA editing activity in tumor epithelial cells, accompanied by alterations in protein metabolism‐related pathways. However, our results extend this framework by revealing that the functional consequences of ADAR1 activity are highly cell‐type dependent. In contrast to epithelial compartments, ADAR1 expression in T cells is tightly coupled to immune exhaustion and aberrant proliferative states, underscoring the necessity of cell‐type‐specific interpretation of ADAR1‐associated signals within the TME.

Building on this insight, we further identified the proliferative CD8^+^ Trm‐mitotic subpopulation as a key immune context in which ADAR1‐associated dysfunction is concentrated. Previous studies have reported that highly proliferative dysfunctional CD8^+^ T cells share transcriptional programs with regulatory T cells across multiple cancer types, including melanoma, non‐small cell lung cancer, and breast cancer [51, 52, 53, 54]. In line with these observations, our data demonstrate that both CD8^+^ Trm‐mitotic and Treg populations exhibit elevated ADAR1 expression, increased inhibitory receptor signaling (including LAG3 and TIGIT), and higher exhaustion scores. Importantly, ADAR1 expression within these populations was markedly reduced following immune checkpoint blockade, highlighting their pivotal role in shaping an immunosuppressive tumor microenvironment. Together, these findings position ADAR1‐associated T cell states as a potential therapeutic vulnerability and suggest that targeting ADAR1 within the CD8^+^ Trm‐mitotic subpopulation may represent a novel immunotherapeutic strategy.

Several limitations of this study should be acknowledged. Although full‐length single‐cell RNA sequencing enabled accurate detection of RNA editing events, its limited throughput constrained the scalability and broader applicability of our analyses. Although bioinformatic, functional, in vivo, and multi‐cohort immunotherapy analyses collectively support the relevance of ADAR1 in T cells, the available clinical cohorts remain limited in size and retrospective in nature. Larger prospective studies, deeper mechanistic dissection of downstream editing and RNA‐sensing pathways, and cross‐tumor validation will be needed.

In conclusion, our study provides a single‐cell framework for understanding RNA editing heterogeneity in CRC and identifies ADAR1 as a key regulator of dysfunctional T cell states associated with resistance to immune checkpoint blockade. By linking ADAR1 activity to immunosuppressive signaling programs such as TGF‐β‐SMAD activation, these findings highlight the cell‐specific functions of ADAR1 and suggest that T cell‐targeted modulation of ADAR1 may offer a promising strategy to sensitize CRC patients to immunotherapy.

## 5 Conclusion

Our study identifies an immune‐intrinsic mechanism by which ADAR1 promotes T cell dysfunction in colorectal cancer. Elevated ADAR1 activity drives T cell exhaustion and impairs antitumor immunity, at least in part through activation of the TGF‐β‐SMAD signaling pathway. Functional and in vivo experiments confirm that ADAR1 limits T cell‐mediated tumor control. Across immunotherapy cohorts and clinical specimens, increased ADAR1 in tumor‐infiltrating T cells is associated with poor response to anti‐PD‐1 therapy, highlighting its potential as a therapeutic target and biomarker.

## Author Contributions

**Da Kang**: Conceptualization, Methodology, Formal analysis, Investigation, Data Curation, Writing – Original Draft, Writing – Review & Editing, Visualization. **Song‐Zuo Xie**: Methodology, Formal analysis, Writing – Original Draft, Visualization. **Yong‐Zhou Luo, Xin‐Pei Deng**: Data Curation, Investigation, Visualization. **Xi‐Rong Tan, Ze‐Geng Chen**: Methodology, Formal analysis. **Ling‐Xing Zeng**: Conceptualization, Methodology. **Gong Chen**: Conceptualization, Validation, Resources. **Zhi‐Zhong Pan, Pei‐Rong Ding**: Methodology, Conceptualization, Resources, Funding acquisition. **Rong‐Xin Zhang**: Conceptualization, Methodology, Resources, Writing – Review & Editing, Supervision, Project administration, Funding acquisition.

## Funding

This work was supported by grants from the National Natural Science Foundation of China (32070592 to Jia‐Xing Yue) and the Guangdong Basic and Applied Basic Research Foundation (2023A1515010243 to Rong‐Xin Zhang).

## Ethics Statement

This study was carried out according to the guidelines of the Declaration of Helsinki and approved by the Ethics Committees of Sun Yat‐Sen University Cancer Center (NO. B2024‐866‐01). The clinical specimens were retrospectively collected and de‐identified before analysis. The requirement for written informed consent was waived by the Ethics Committee because of the retrospective design, use of de‐identified specimens, and minimal risk to participants. This study was registered in the national medical research registration system (https://www.medicalresearch.org.cn/).

## Conflicts of Interest

The authors declare no conflicts of interest.

## Supporting information




**Supporting File 1**: advs76143‐sup‐0001‐SuppMat.docx.

**Supporting File 2**: smll72608‐sup‐0002‐TableS1.xlsx.

**Supporting File 3**: smll72608‐sup‐0003‐TableS2.xlsx.

## Data Availability

Data sharing not applicable to this article as no datasets were generated or analyzed during the current study.
