# Supplementary material for: Single‐Cell RNA Editing Identifies T Cell ADAR1 as a Key Regulator of Immune Exhaustion and Anti‐PD‐1 Resistance in Colorectal Cancer
Source: Adv Sci (Weinh). 2026 Jun 15:e76143. Online ahead of print. doi: 10.1002/advs.76143 (PMC13336568; doi:10.1002/advs.76143)
Supplement: Supplementary file 1 — Supporting File 1: advs76143‐sup‐0001‐SuppMat.docx. [file ADVS-9999-e76143-s003.docx]

**Supplementary Figure 1**


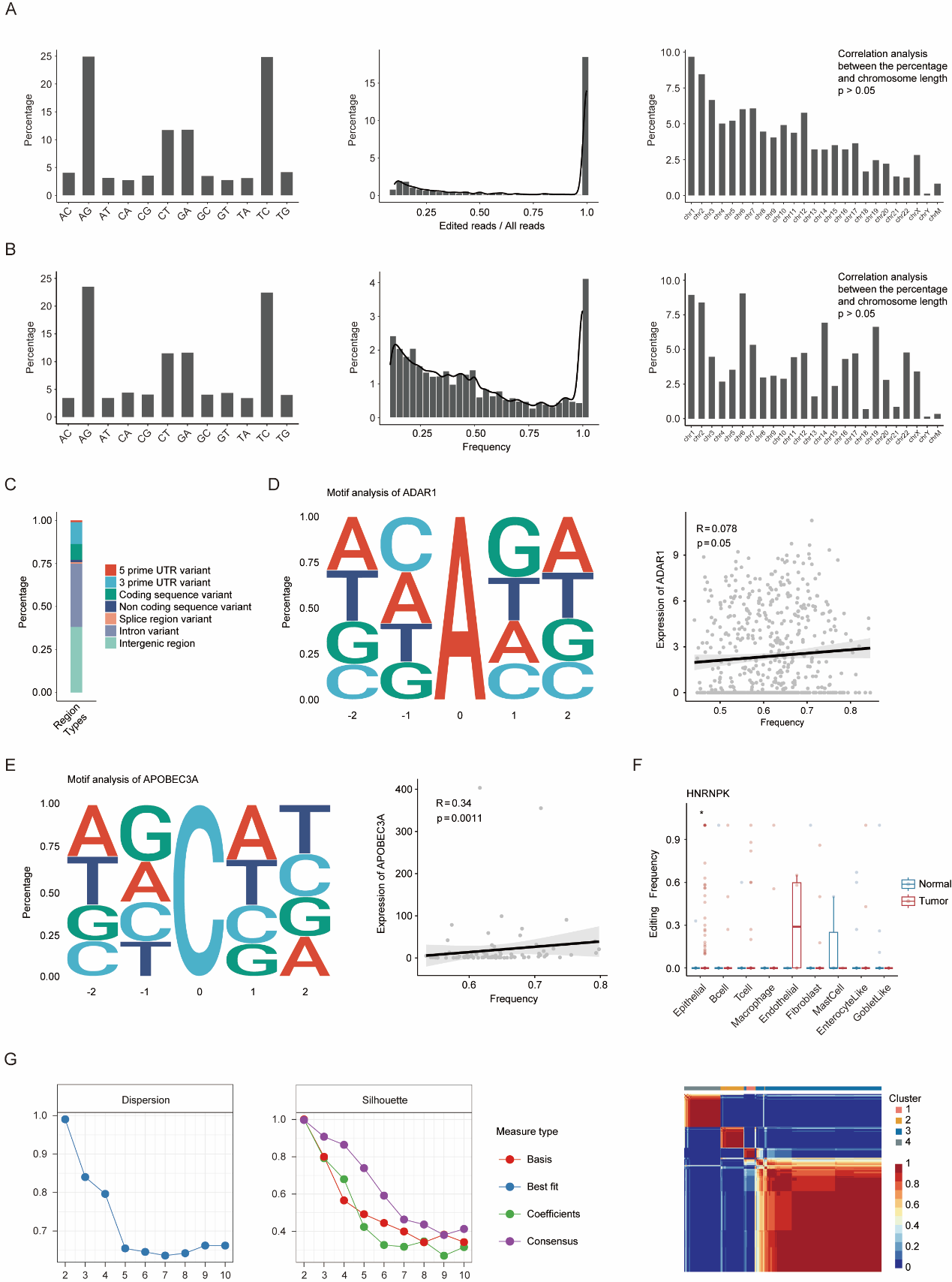


**A-B,** Distribution of RNA editing site types, editing frequencies, and chromosomal distributions in the EGAD00001002727 sc-RNA-seq dataset (A) and the TCGA-CRC bulk RNA-seq dataset (B). Correlation analyses between the proportion of RNA editing sites and chromosome length are shown, with no significant correlations observed. **C,** Genomic annotation of RNA editing sites. **D,** Motif analysis of A-to-I RNA editing sites and correlation between A-to-I editing frequency and ADAR1 expression. **E,** Motif analysis of C-to-U RNA editing sites and correlation between C-to-U editing frequency and APOBEC3A expression. **F,** Editing frequencies of HNRNPK across different cell types and tissues. **G,** NMF-based clustering of RNA-SBS signatures, identifying four distinct editing signature clusters. *, P value < 0.05; **, P value < 0.01; ***, P value < 0.001; ****, P value < 0.0001.

**Supplementary Figure 2**


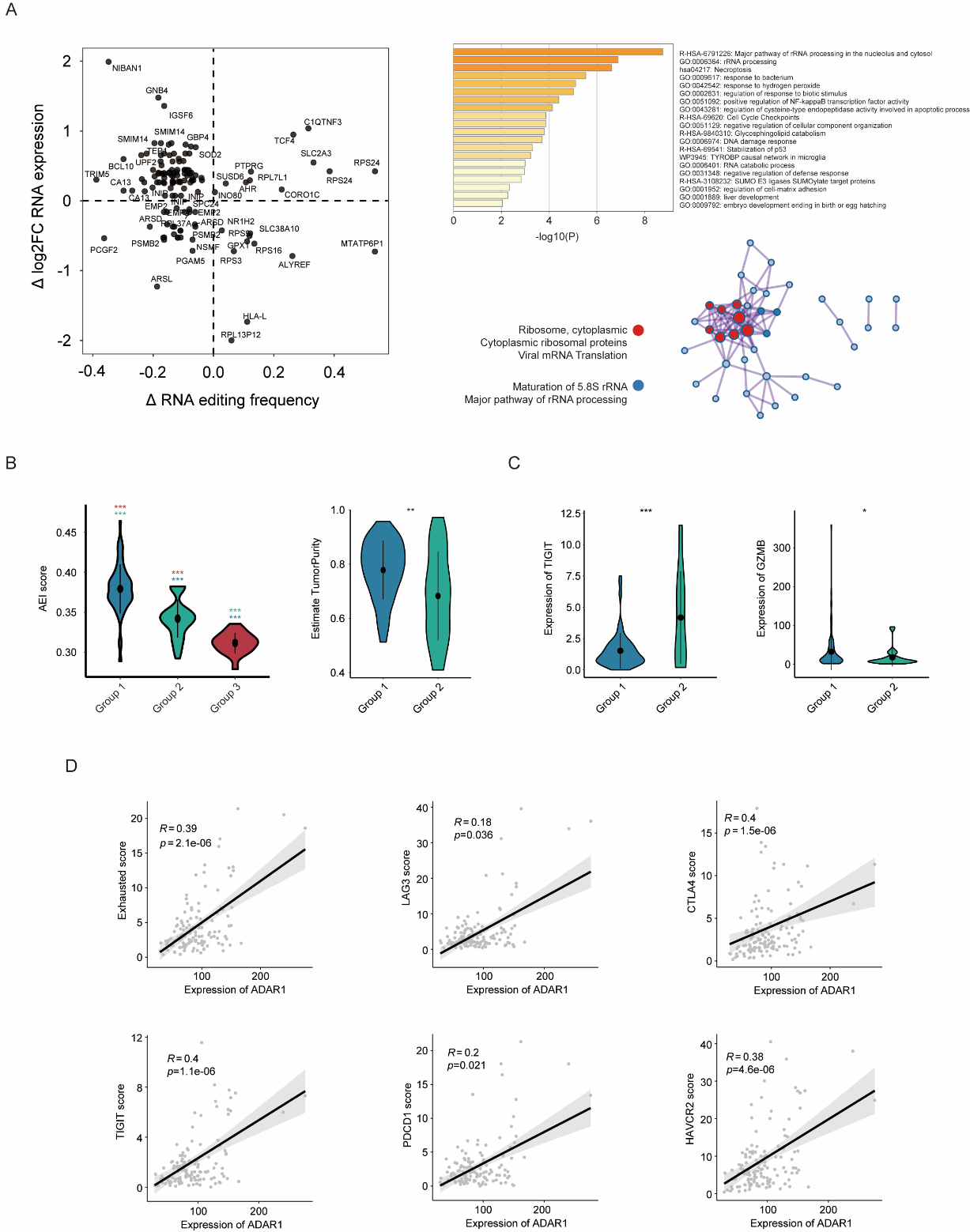


**A,** Left, relationship between changes in RNA editing frequency and corresponding changes in gene expression for coding genes in the TCGA-CRC cohort. Right, functional enrichment analyses of edited genes, highlighting RNA processing- and translation-related pathways. **B,** Comparison of the AEI and ESTIMATE tumor purity scores across RNA editing-defined clustering groups. **C,** Expression levels of representative function-associated genes in group 1 and group 2. **D,** Correlation analyses between ADAR1 expression and exhausted markers in TCGA-CRC samples. *, P value < 0.05; **, P value < 0.01; ***, P value < 0.001; ****, P value < 0.0001.

**Supplementary Figure 3**


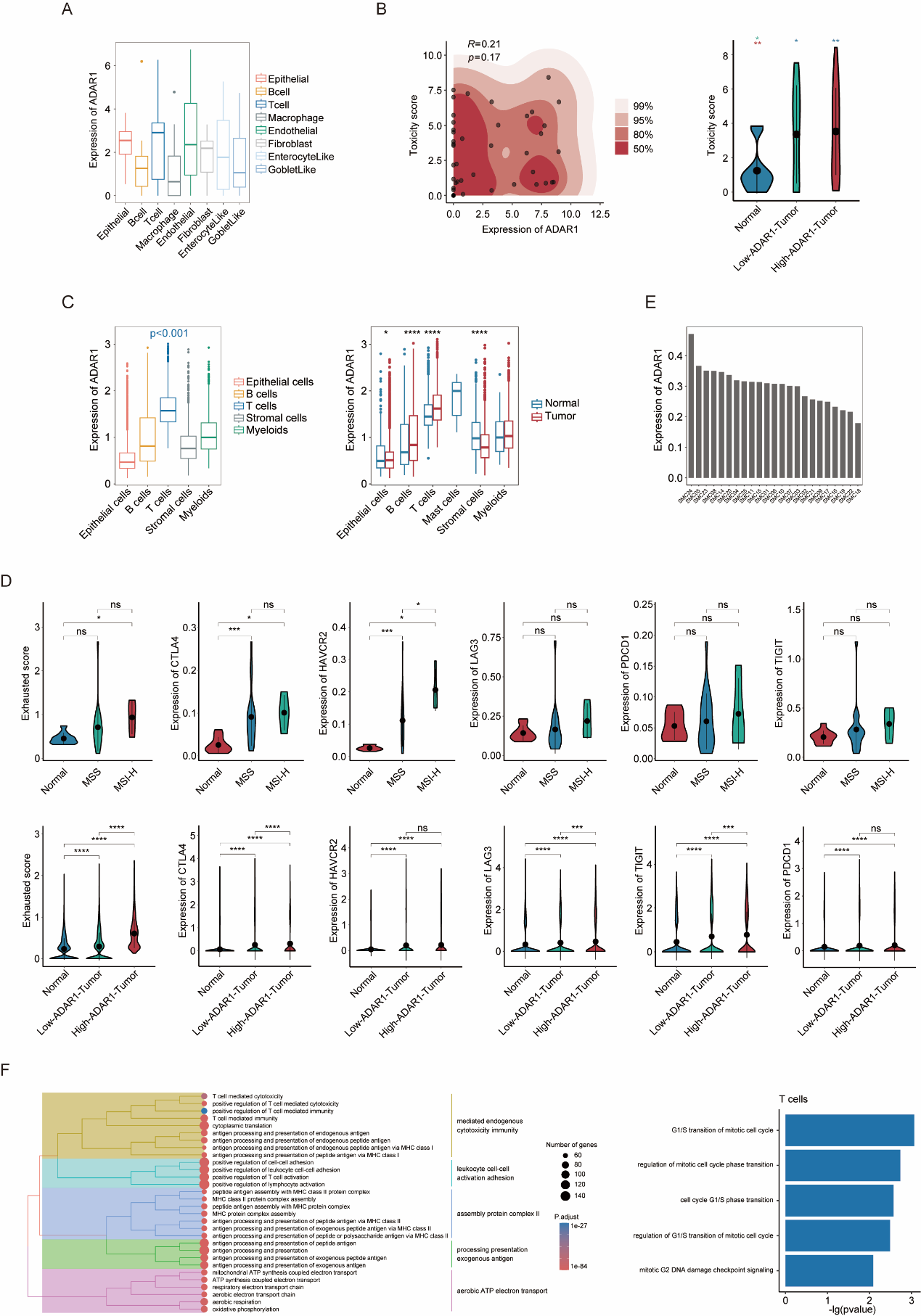


**A,** ADAR1 expression levels across major cell subtypes in the EGAD00001002727 full-length scRNA-seq dataset. **B**, Association between ADAR1 expression and T cell functional scores in the EGAD00001002727 dataset. **C,** ADAR1 expression levels across major cell lineages and comparison between normal and tumor tissues in the GSE132465 scRNA-seq dataset. **D,** Exhaustion scores and expression of exhaustion-associated markers (CTLA4, HAVCR2, LAG3, PDCD1, and TIGIT) stratified by microsatellite status and by ADAR1 expression levels in the GSE132465 dataset. **E,** Patients ranked by average ADAR1 expression across tumor samples in the GSE132465 dataset. **F,** Functional enrichment analysis of ADAR1-associated genes in T cells from the GSE132465 dataset, highlighting pathways related to immune activation, antigen presentation, and cell-cycle regulation. *, P value < 0.05; **, P value < 0.01; ***, P value < 0.001; ****, P value < 0.0001.

**Supplementary Figure 4**


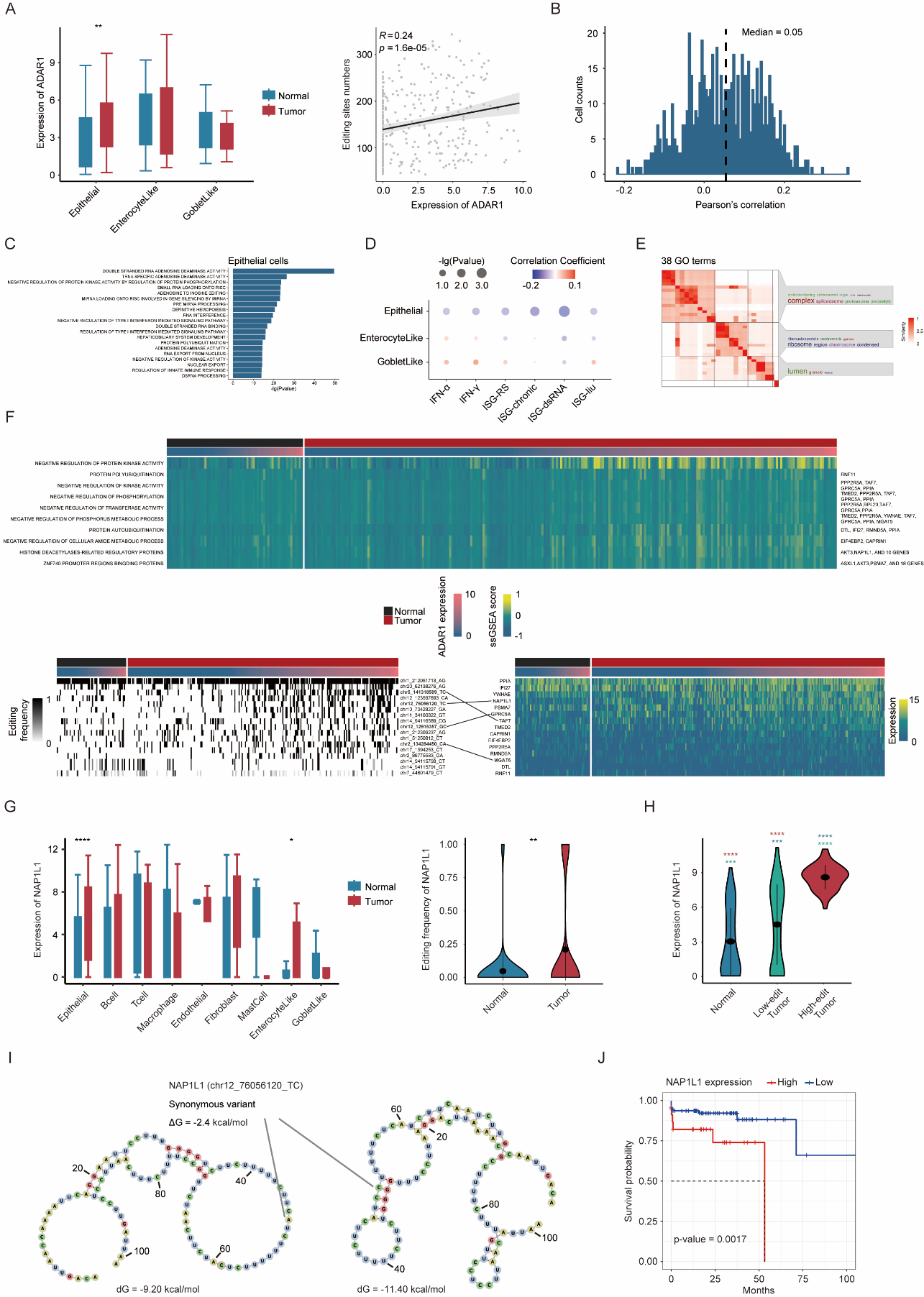


**A,** ADAR1 expression levels in epithelial cell subsets from normal and tumor tissues, and correlation between ADAR1 expression and the number of RNA editing sites per cell. **B,** Distribution of Pearson correlation coefficients between ADAR1 expression and RNA editing frequency across epithelial cells. **C,** Enrichment analysis of ADAR1-associated genes in epithelial cells, highlighting pathways related to protein metabolism and RNA processing. **D,** Correlation analysis between ADAR1 expression and IFN-related gene signatures across epithelial and epithelial-like cell populations. **E,** Heatmap of enriched terms based on ADAR1-associated transcriptional programs in epithelial cells. **F,** Integrated heatmaps showing ssGSEA scores, RNA editing frequencies, and expression levels of protein metabolism-related genes in epithelial cells, stratified by tissue type. **G,** Expression levels and RNA editing frequencies of NAP1L1 across different cell types and tissue conditions. **H,** NAP1L1 expression levels in normal tissues and in tumors stratified by ADAR1 expression status. **I,** RNA secondary structure prediction of NAP1L1 transcripts, showing reduced free energy in the edited RNA compared with the unedited form. **J,** Kaplan-Meier survival analysis of CRC patients stratified by NAP1L1 expression levels. *, P value < 0.05; **, P value < 0.01; ***, P value < 0.001; ****, P value < 0.0001.

**Supplementary Figure 5**


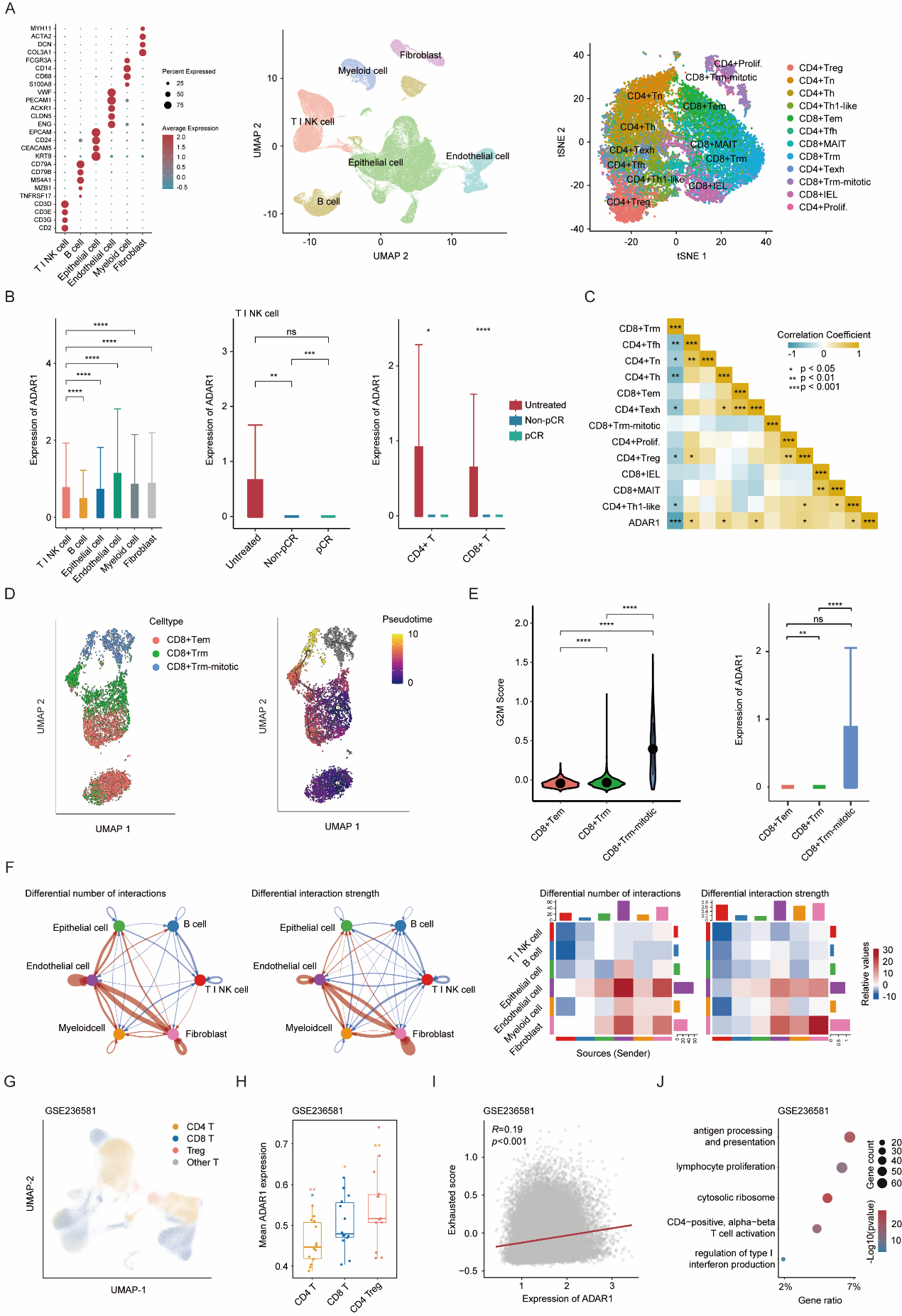


**A,** Canonical marker expression and dimensionality reduction used to annotate major cell types and T cell subpopulations in the GSE205506 scRNA-seq dataset. **B,** ADAR1 expression levels across major cell types and T cell subsets, stratified by treatment status (untreated, non-pCR, and pCR). **C,** Correlation analysis between bulk ADAR1 expression and the relative abundance of T cell subpopulations across patients. **D,** Pseudotime trajectory analysis depicting differentiation relationships among CD8⁺ T cell subsets, including CD8⁺ Tem, CD8⁺ Trm, and CD8⁺ Trm-mitotic cells. **E,** Cell-cycle (G2/M) scores and ADAR1 expression levels across CD8⁺ T cell subpopulations. **F,** Cell-cell communication analysis comparing interaction number and interaction strength between ADAR1-high and ADAR1-low T cells with other immune and stromal populations. **G**, Dimensionality reduction of T cell populations in the GSE236581 cohort, showing major T cell compartments. **H**, Mean ADAR1 expression levels across major T cell compartments in the GSE236581 cohort. **I**, Correlation analysis between ADAR1 expression and exhaustion scores in T cells from the GSE236581 cohort. **J**, Functional enrichment analysis of ADAR1-high T cells in the GSE236581 cohort, showing enrichment of interferon-related and antigen presentation-associated programs. *, P value < 0.05; **, P value < 0.01; ***, P value < 0.001; ****, P value < 0.0001.

**Supplementary Figure 6**


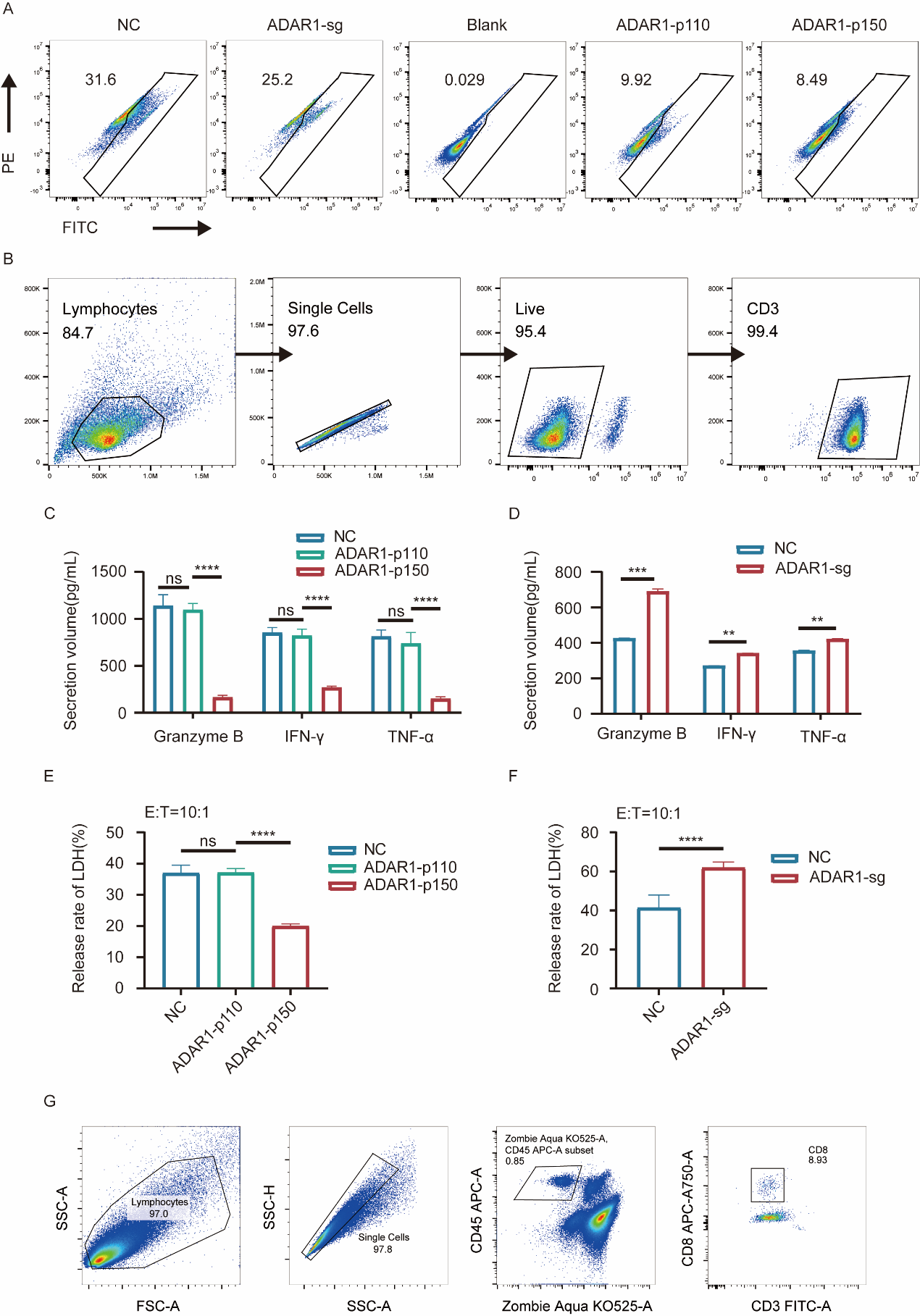


**A,** Flow cytometric assessment of transduction efficiency in human primary T cells transduced with negative control (NC), ADAR1-overexpressing, or ADAR1-knockout constructs. **B,** Flow cytometric gating strategy used for in vitro T cell functional assays. **C-D,** ELISA-based quantification of Granzyme B, IFN-γ, and TNF-α levels in coculture supernatants from ADAR1-overexpressing (C) or ADAR1-knockout (D) T cells. **E-F,** LDH release assays measuring cytotoxic activity of ADAR1-overexpressing (E) or ADAR1-knockout (F) T cells against HCT116 colorectal cancer cells. **G,** Flow cytometric gating strategy used for the analysis of tumor-infiltrating immune cells in vivo. *, P value < 0.05; **, P value < 0.01; ***, P value < 0.001; ****, P value < 0.0001.

**Supplementary Figure 7**


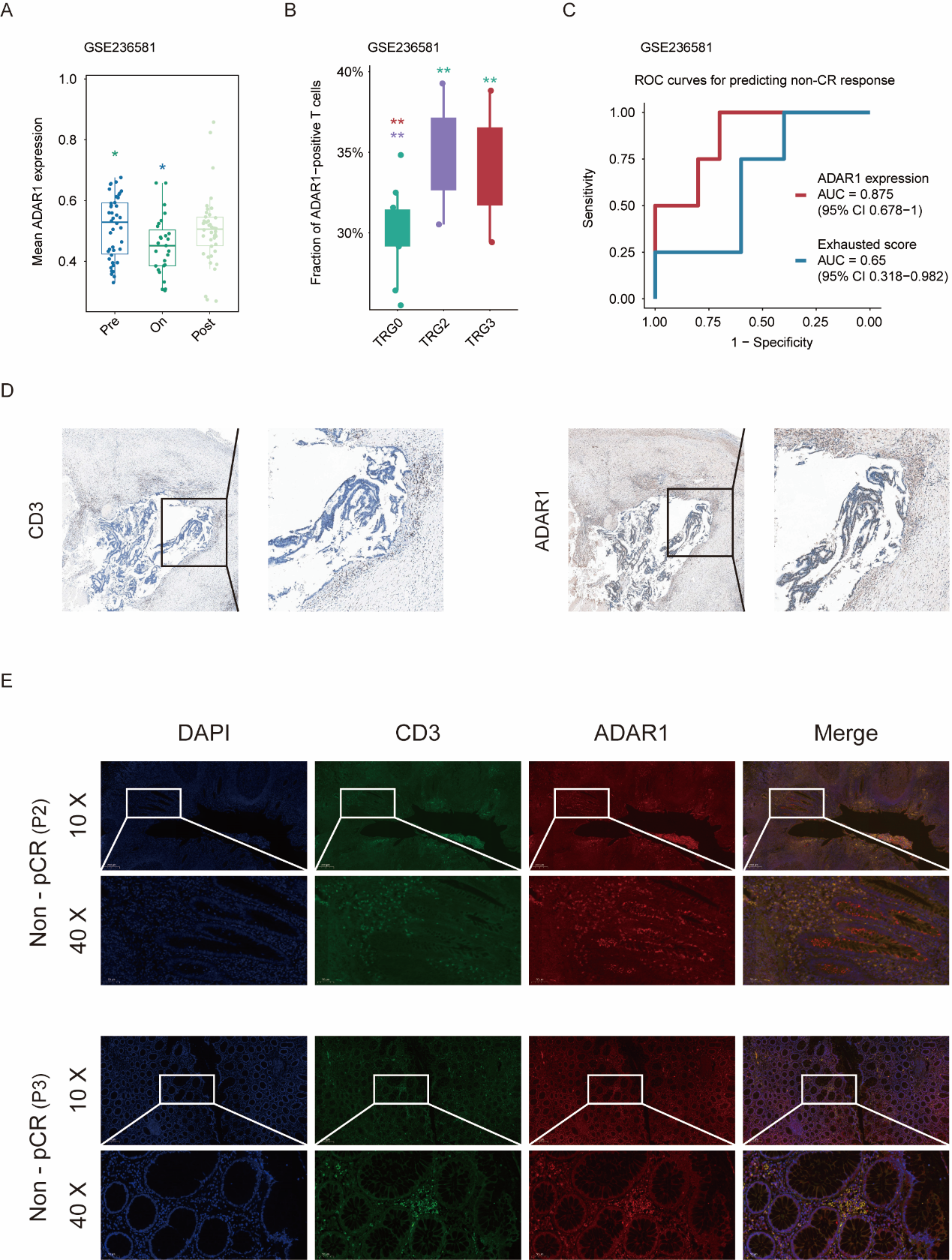


**A**, Mean ADAR1 expression in T cells before, during, and after ICI treatment in the GSE236581 cohort. **B**, Fraction of ADAR1-positive T cells in patients stratified by tumor regression grade (TRG) in the GSE236581 cohort. **C**, ROC curves comparing T cell ADAR1 expression and exhaustion score for distinguishing CR from non-CR patients. AUC values and 95% confidence intervals are shown. **D**, Representative staining of CD3 and ADAR1 in paired CRC tumor sections, showing spatial localization of ADAR1 relative to CD3⁺ T cells. **E**, Representative multiplex immunofluorescence staining of CRC tumor sections from non-pCR patients with ICI treatment, showing DAPI, CD3, ADAR1, and merged images. *, P value < 0.05; **, P value < 0.01; ***, P value < 0.001; ****, P value < 0.0001.
